# Odorant receptors for floral- and plant-derived volatiles in the yellow fever mosquito, *Aedes aegypti* (Diptera: Culicidae)

**DOI:** 10.1371/journal.pone.0302496

**Published:** 2024-05-06

**Authors:** Heidi Pullmann-Lindsley, Robert Mark Huff, John Boyi, Ronald Jason Pitts

**Affiliations:** Department of Biology, Baylor University, Waco, TX, United States of America; USDA Agricultural Research Service, UNITED STATES

## Abstract

Adult mosquitoes require regular sugar meals, including nectar, to survive in natural habitats. Both males and females locate potential sugar sources using sensory proteins called odorant receptors (ORs) activated by plant volatiles to orient toward flowers or honeydew. The yellow fever mosquito, *Aedes aegypti* (Linnaeus, 1762), possesses a large gene family of ORs, many of which are likely to detect floral odors. In this study, we have uncovered ligand-receptor pairings for a suite of *Aedes aegypti* ORs using a panel of environmentally relevant, plant-derived volatile chemicals and a heterologous expression system. Our results support the hypothesis that these odors mediate sensory responses to floral odors in the mosquito’s central nervous system, thereby influencing appetitive or aversive behaviors. Further, these ORs are well conserved in other mosquitoes, suggesting they function similarly in diverse species. This information can be used to assess mosquito foraging behavior and develop novel control strategies, especially those that incorporate mosquito bait-and-kill technologies.

## Introduction

Arthropods rely on sensory receptors to interpret and interact with their environments. Arguably, olfaction is the most important sensory modality, conveying the ability to sense volatile chemical cues over large distances [1–3]. Olfaction is responsible for many arthropod behaviors, including host-seeking [4, 5], oviposition [6, 7], mate selection [8, 9], and nectar foraging [10, 11]. Odorant receptors (ORs) comprise the most prominent family of volatile odor detectors encoded in mosquito genomes [12]. Another family of proteins called ionotropic receptors (IRs) also functions in insect olfaction, especially in organic acid detection [13, 14]. ORs are transmembrane proteins expressed by odorant receptor neurons housed in hairlike projections called sensilla [15, 16]. Sensilla sensory appendages such as antennae, maxillary palps, labella, and leg tarsi constitute the primary olfactory appendages in insects [17–19]. ORs are ligand-gated ion channels that respond to diverse, environmentally relevant volatile odorants [20]. ORs most likely form multimers that include co-expression of a single type of tuning OR complexed with an odorant receptor coreceptor (Orco), which is highly conserved across most insect taxa [20–22]. Chemical volatiles enter the sensilla through tiny pores on their surfaces and either diffuse through the sensilla lymph or are shuttled by odorant binding proteins to the neuronal membrane, where they can interact with the OR/Orco complex [23, 24]. Generally, single ORs respond to one compound most robustly, though they may demonstrate the responsiveness of several other ligands. Most arthropod species have between 50 and 100 different ORs, which allow for complex and robust interactions with their respective environments through the perception of various volatile compounds. However, some species have as few as five or as many as 300 [25, 26]. The diversity of receptor proteins underlies distinct behavioral phenomena across arthropod species.

By investigating the molecular intricacies of how these receptors function, we can contribute to our understanding of insect chemical ecology, especially for mosquitoes that vector human pathogens. Deorphanizing single ORs has yielded valuable insights into many insect species, directly informing behavioral control strategies. Moth pheromones are examples of some of the most comprehensively studied ligand-OR relationships to date [27]. In the silk moth, *Bombyx mori*, BmOR1, responded with high specificity to the female-produced pheromone bombykol [28, 29] using a heterologous expression system [30]. Notably, disrupting BmOR1 led to the complete loss of sensitivity to bombykol, subsequently impacting pheromone source detection in male moths [31]. A pheromone and accompanying receptor have also been characterized in the cotton leafworm, *Spodoptera littoralis* [32, 33]. As in *Bombyx mori*, the disruption of the pheromone receptor yielded a decreased response to the pheromone [34], and the knock-out of the co-receptor led to defects in mate-seeking behavior [35]. While the intricate relationships between pheromones, ORs, and mating behavior are highly species-specific, ORs are linked to several other critical behaviors, including nectar foraging in vector mosquitoes. Understanding their function could prove useful in improving existing surveillance methods, or designing new control strategies.

*Aedes aegypti* (Linnaeus, 1762) is a prominent disease vector that transmits several arboviruses including Dengue, Zika, and yellow fever across much of Africa, Asia, South America, Oceania, and parts of North America and Europe [36]. As climate change continues to alter ecosystems and global urbanization accelerates, the viable range of *Ae*. *aegypti* may also expand, potentially increasing its threat to human health [37, 38]. Despite the significant impact of this species, relatively few of the >125 ORs that are encoded in the *Ae*. *aegypti* genome and expressed in various tissues and life stages have been functionally characterized [39–43]. Studies have reported ORs presumed to contribute to female host-seeking behaviors and single sensillum recordings (SSR) have revealed that *Ae*. *aegypti* neurons responded to a variety of chemical compounds collected from human volunteers, including octanal and nonanal [44–46]. Another study demonstrated that a highly conserved OR in Culicidae responds to carboxylic acids present on human skin [5]. Despite this progress, insights into the molecular aspects of volatile odor detection for other essential behaviors like floral odor detection across divergent species like *Ae*. *aegypti* and *Anopheles gambiae* (Giles 1902), are generally lacking, especially for larger sets of ORs. Remarkably, little is known about the shared behavior of nectar foraging between males and females, which is crucial for mosquito survival and reproductive fitness [47]. Mosquitoes inhabit a variety of ecological settings in which they must be able to adapt to local conditions to accurately respond to odors associated with flowers for nectar foraging. While preference between nectar sources has been documented in numerous mosquito species, the sensory underpinnings are yet to be thoroughly elucidated, particularly in *Ae*. *aegypti* [48–51]. Recent studies have begun to characterize receptors that may be linked to floral odor sensing in mosquitoes [43], yet a more comprehensive set of OR-ligand profiles is warranted. Characterizing the ORs that mediate the detection of plant-derived compounds may provide vital information about nectar-seeking attractants that can be used in mosquito bait-and-kill systems (MBAKS) or attractive toxic sugar baits (ATSBs) [52–54]. While MBAKS and ATSBs have demonstrated relative success in reducing mosquito populations [55], pursuing a more specific attraction to mosquito species could reduce off-target effects [56]. The present study aims to expand upon both the number of receptors characterized and the number of chemical compounds tested.

In this study, we investigated ORs in *Ae*. *aegypti* that are conserved in several disease vector mosquito species and are closely related to other ORs that function in floral odor sensing [43, 57, 58]. These receptors are expressed mainly in male and female antennae [39, 40, 42], suggesting they may function in a shared behavior such as nectar foraging. We initiated this study by combining manual gene annotations with heterologous expression and two-electrode voltage clamping (TEVC) to identify activating ligands from a library of 147 chemical compounds [59–61]. Using this method, we characterized ORs in *Ae*. *aegypti* to improve our understanding of the molecular basis for mosquito nectar seeking or other vital behaviors.

## Materials and methods

### Phylogenetic analysis and gene annotations

A conserved clade of genes appears to be activated by floral or nectar odors and utilized by mosquitoes in foraging. These genes were identified via BLAST via tBLASTn or BLASTp using VectorBase and confirmed using available RNAseq data (S1 and S2 Files). Closely related ORs from *Aedes albopictus* (Skuse, 1894) and *Culex quinquefasciatus* (Say, 1823) were also identified (S3 and S4 Files). DNA sequences from the reference genomes (*Ae*. *aegypti* LVP_AGWG, *Ae*. *albopictus* Foshan FPA, *Cx*. *quinquefasciatus* JHB 2020) were downloaded and the genes were manually annotated using SnapGene (Dotmatics, Boston, MA, USA). Intron analysis was conducted for the *Ae*. *aegypti* genes and compared with previously identified conserved introns [39]. A Neighbor-Joining tree was constructed sing Geneious Prime software (Biomatters Ltd., Boston, MA, USA) from a multiple alignment of amino acids for homologous receptors.

### Gene cloning and sequencing

The coding regions of ten AaegOR genes of interest (S3 File) and AaegOrco were synthesized by Twist Bioscience (San Francisco, CA, USA) and cloned into the pENTR plasmid. The Gateway LR directional cloning system (Invitrogen Corp., Carlsbad, CA, USA) was utilized to subclone AaegOR coding regions into the pSP64t-RFA oocyte expression plasmid. Plasmids were purified using the GeneJet Plasmid Miniprep Kit (ThermoFisher Scientific, Waltham, MA, USA). Purified plasmids were sequenced in both directions to confirm coding regions.

### OR gene expression analysis and de novo transcript assembly

OR expression values in Transcripts Per Million (TPM) were obtained from VectorBase (vectorbase.org) using data from a published study of *Ae*. *aegypti* chemosensory appendages (S5 File) [42]. *De novo* transcripts were assembled using non-bloodfed female antennae and maxillary palp RNAseq data sets [42, 62] using the Trinity software package [41] on the Galaxy server (usegalaxy.org) and aligned using ClustalW with character counts (S2 File) [63].

### Chemical reagents

Chemical compounds (S6 File) used in the deorphanization of AaegORs were purchased from Acros Organics (Morris, NJ, USA), Alfa Aesar (Ward Hill, MA, USA), and ThermoFisher Scientific (Waltham, MA, USA) at the highest purity available. Compounds were stored as 1M stocks in 100% DMSO. Serial dilutions of each compound were prepared in 10% DMSO and blended by chemical class.

### Two-electrode voltage clamping

pSP64t-RFA: AaegOR clones were linearized using Xbal, and cRNAs were synthesized using the mMESSAGE mMACHINE ® SP6 kit (Life Technologies, Carlsbad, CA, USA). *Xenopus laevis* stage V-VII oocytes purchased from Xenopus1 (Dexter, MI, USA) were stored at 18°C in Incubation medium (ND96 96 with 5% dialyzed horse serum, 50μg/mL gentamycin, 100μg/mL streptomycin, 100μg/mL penicillin, and 550μg/mL sodium pyruvate). 30nL of cRNA was injected into multiple oocytes using a microinjection syringe pump (World Precision Instruments, Sarasota, FL, USA) maintained at 18°C for 48–72 hrs. Odorant blends were perfused across individual oocytes for 10–15 seconds, held at a resting membrane potential of -80 mV using an OC-725C oocyte clamp (Warner Instruments, LLC, Hamden, CT, USA). Induced currents were measured indirectly using the two-electrode voltage clamp technique (TEVC). Following each stimulus, currents were allowed to return to baseline before further stimulation. Data was captured using the Digidata 1550 B digitizer and PClamp10 software (Molecular Devices, San Jose, CA, USA). Concentration-response curves were generated by perfusing unitary odorants over oocytes at increasing concentrations from [10^-3^M] to [10^-9^M]. Data were analyzed using GraphPad Prism 8 (GraphPad Software Inc., La Jolla, CA, USA). At least two biological replicates were conducted at each concentration and for each compound. Data for at least seven oocytes responding to each compound was analyzed (S7 File).

## Results

### 1. *Aedes aegypti* odorant receptors are expressed in male and female antennae and conserved in other disease vector mosquitoes

**Fig 1:**
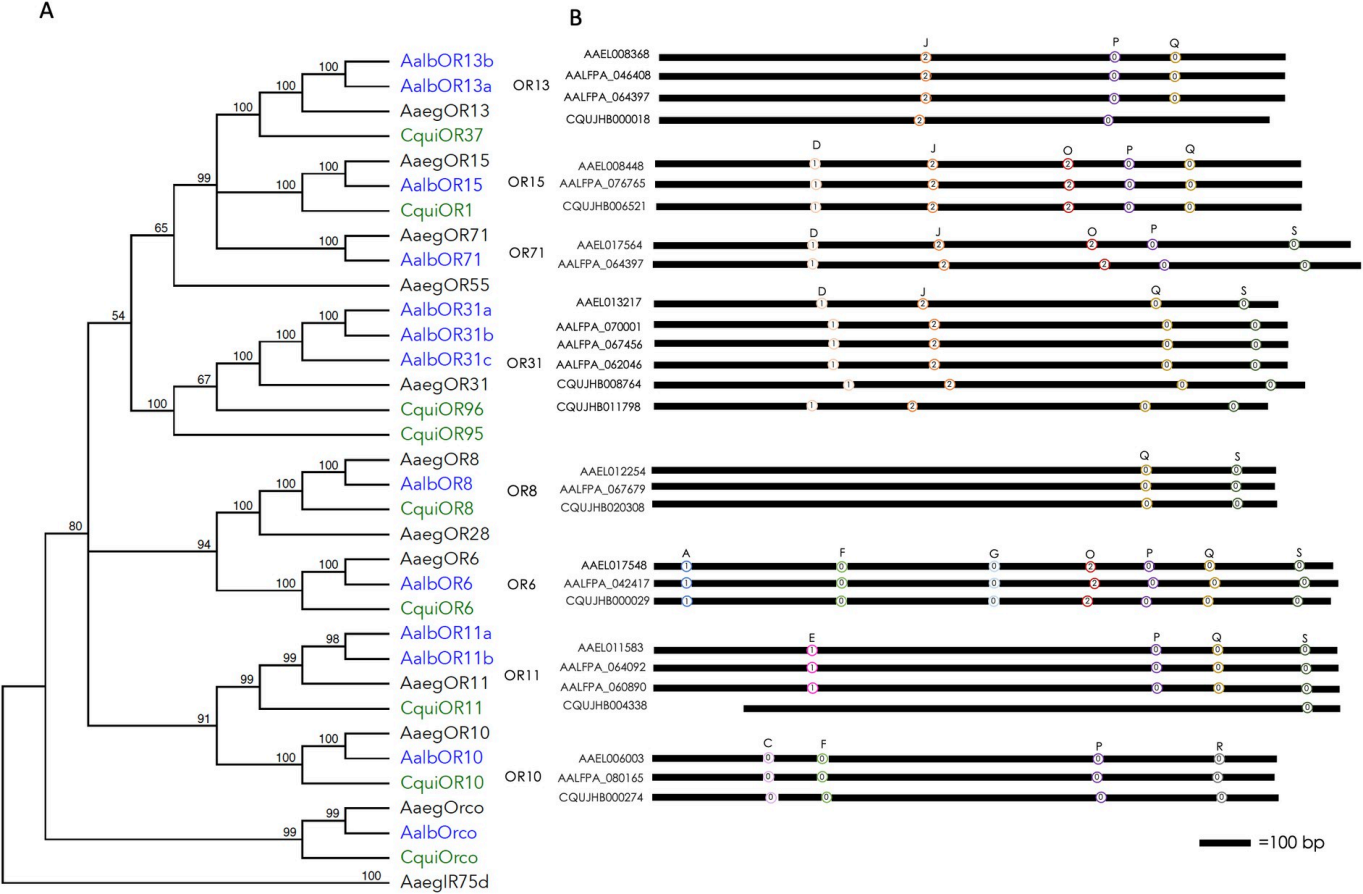
Phylogenetic analysis of candidate floral volatile sensing ORs in major disease vectors. (A) Neighbor-joining tree showing relationships among floral volatile sensing ORs for three disease vector species of mosquitoes: *Ae*. *aegypti* (black), *Ae*. *albopictus* (Blue), and *Cx*. *quinquefasciatus* (green). Bootstrap values from 1000 pseudoreplicates are given at each node. (B) Odorant receptor gene structures. Circles indicate introns, numbers within circles are intron phases. Letters and colors mark conserved positions accoding to [39].

A comparison of sequences used in this study against publicly available RNAseq datasets [40] confirmed AaegOR gene structures (S1 File) and quantified expression of transcrips in adult sensory appendages (Table 1 and S5 File). AaegORs are most abundant in the antennae of both sexes, with the exception of AaegOR8, which is expressed in the maxillary palps (Table 1) [39, 42]. Phylogenetic reconstrcutions using conceptual translations of candidate floral volatile sensing odorant receptors in three major disease vectors, *Ae*. *aegypti*, *Ae*. *albopictus* and *Cx*. *quinquefasciatus*, revealed conserved lineages (Fig 1A), while manually curated gene structures (Fig 1B) and amino acid alignments (S4 File) further support close evolutionary relationships among homologs in Culicinae. In the course of our studies, we identified six new ORs in *Ae*. albopictus, including two OR11 homologs, named AalbOR11a and AalbOR11b, a second OR13 homolog, AalbOR13b, and three previously unannotated OR31 homologs, AalbOR31a, AalbOr31b, AalbOR31c. Overall, our observations provide a subset of ORs that are likely to share similar ligands across multiple mosquito species.

**Table 1:** Transcripts per million in *Ae*. *Aegypti* sensory appendages.

			Transcripts per million (+/- SE) in *Ae*. *aegypti* head appendages					
AaegOR	VectorBase ID	NCBI ID	female ant.	male ant.	female palp	male rost.	female prob.	TPM Scale:
Orco	AAEL005776	NM_001358471	2545.4 (146.6)	385.1 (9.2)	985.6 (87.3)	80.6 (4.2)	35.7 (2.1)	< 1.0
OR6	AAEL017548	NM_001358467	5.3 (1.0)	6.6 (0.5)	0.1 (0.1)	2.6 (0.4)	5.8 (0.7)	1.0>,<10.0
OR8	AAEL012254	XM_001662305.3	0 (0.0)	0 (0.0)	387.7 (37.8)	25.7 (1.3)	0.2 (0.2)	10.0>,<100.0
OR10	AAEL006003	NM_001358339	21.0 (3.0)	11.5 (0.6)	0 (0.0)	0.5 (0.0)	0.4 (0.1)	100.0>,<1000.0
OR11	AAEL011583	NM_001358172	146.1 (8.9)	37.0 (1.1)	0 (0.0)	0.2 (0.1)	2.5 (0.5)	>1000.0
OR13	AAEL008368	NM_001358180	20.9 (1.5)	3.8 (0.6)	0 (0.0)	0.3 (0.0)	0.6 (0.1)	
OR15	AAEL008448	NM_001358181	6.7 (1.2)	3.3 (0.2)	0 (0.0)	2.2 (0.2)	4.0 (0.3)	
OR28	AAEL027053	NM_001358166	4.0 (0.4)	3.4 (0.2)	0 (0.0)	0 (0.0)	0 (0.0)	
OR31	AAEL013217	NM_001358160	19.0 (1.5)	10.4 (0.7)	0.2 (0.1)	0.5 (0.0)	1.0 (0.3)	
OR55	AAEL010415	NM_001358184	4.4 (0.3)	1.4 (0.3)	0 (0.0)	0.5 (0.1)	1.3 (0.5)	
OR71	AAEL017564	NM_001358112	43.1 (2.7)	14.6 (0.3)	0.9 (0.3)	2.9 (0.4)	9.4 (0.7)	

Data from vectorBase.org (Matthews *et al*. 2016)

### 2. AaegORs respond to environmentally relevant compounds in a concentration-dependent manner

**Fig 2:**
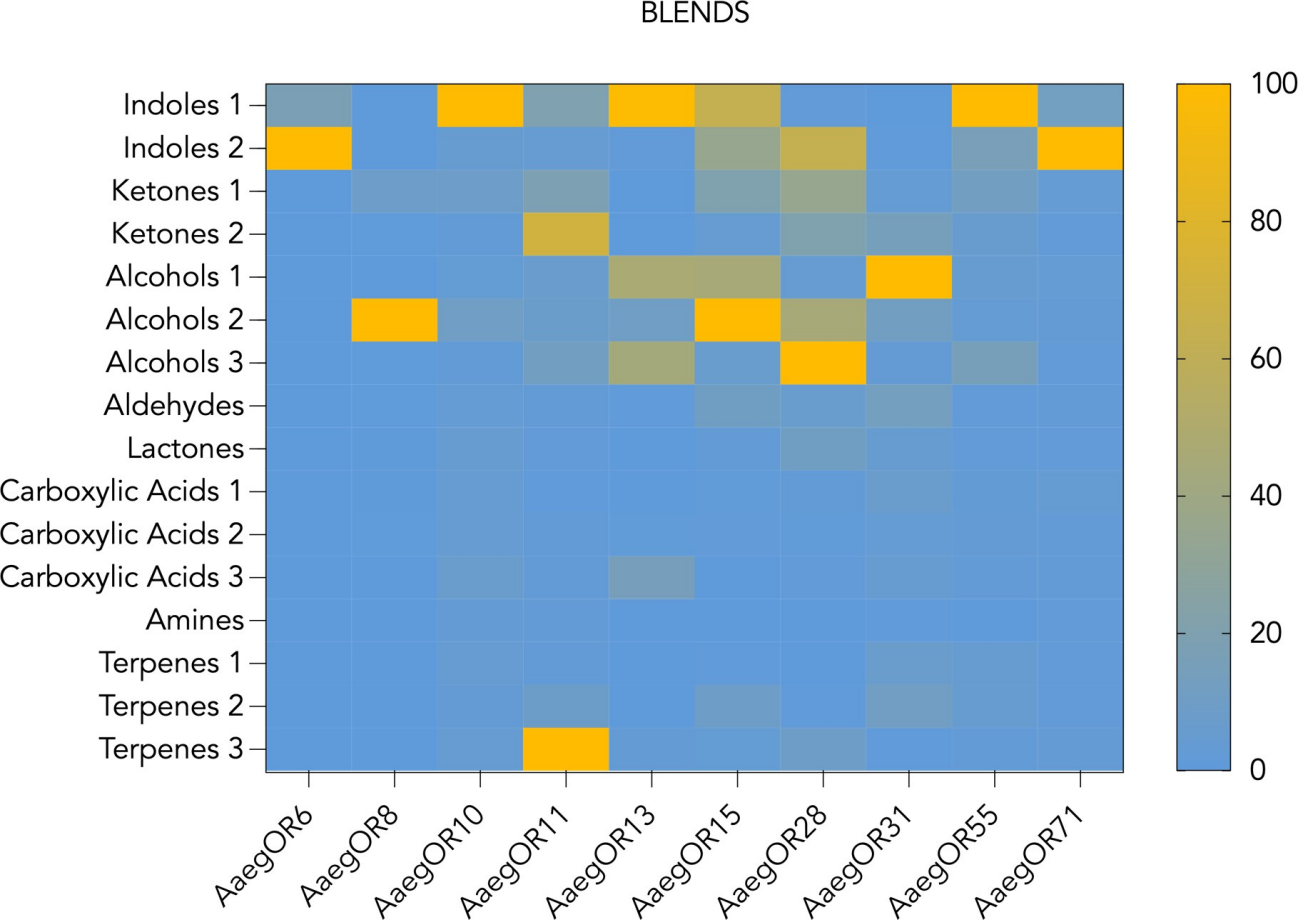
Heat map gradation of normalized AaegOR responses. Blends of chemical compounds used for receptor screening are listed on the left-hand side, while receptor identities are listed as columns below. The scale to the right spans the normalized response range from highest (100, yellow) to lowest (0, blue).

**Fig 3:**
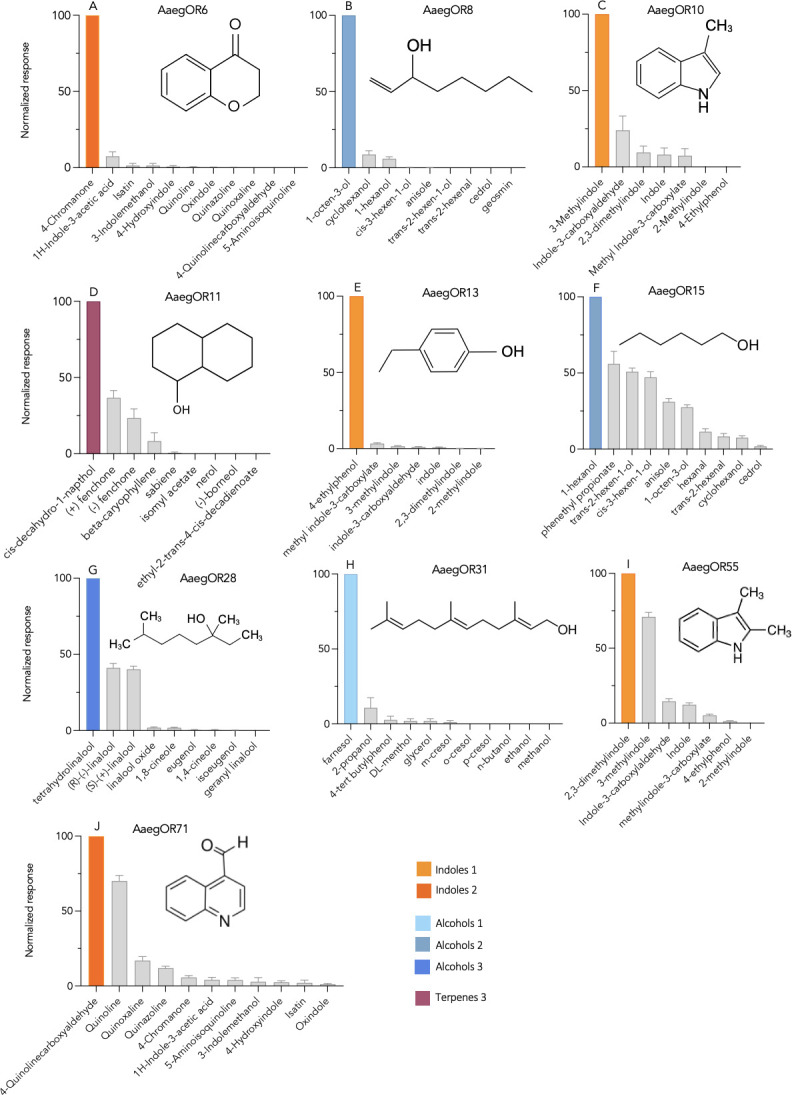
*Ae*. *aegypti* odorant receptor responses to blend components. Normalized mean current responses of AaegORX/Orco expressing oocytes (n = 7–10) to individual chemical compounds. (A) 4-chromanone, (B) 1-octen-3-ol, (C) 3-methylindole, (D) cis-decahydro-1-napthol, (E) 4-ethylphenol, (F) 1-hexanol, (G) tetrahydrolinalool, (H) farnesol, (I) 2,3-dimethylindole, and (J) 4-quinolinecarboxyaldehyde. The structure of each compound is shown in figure inserts.

**Fig 4:**
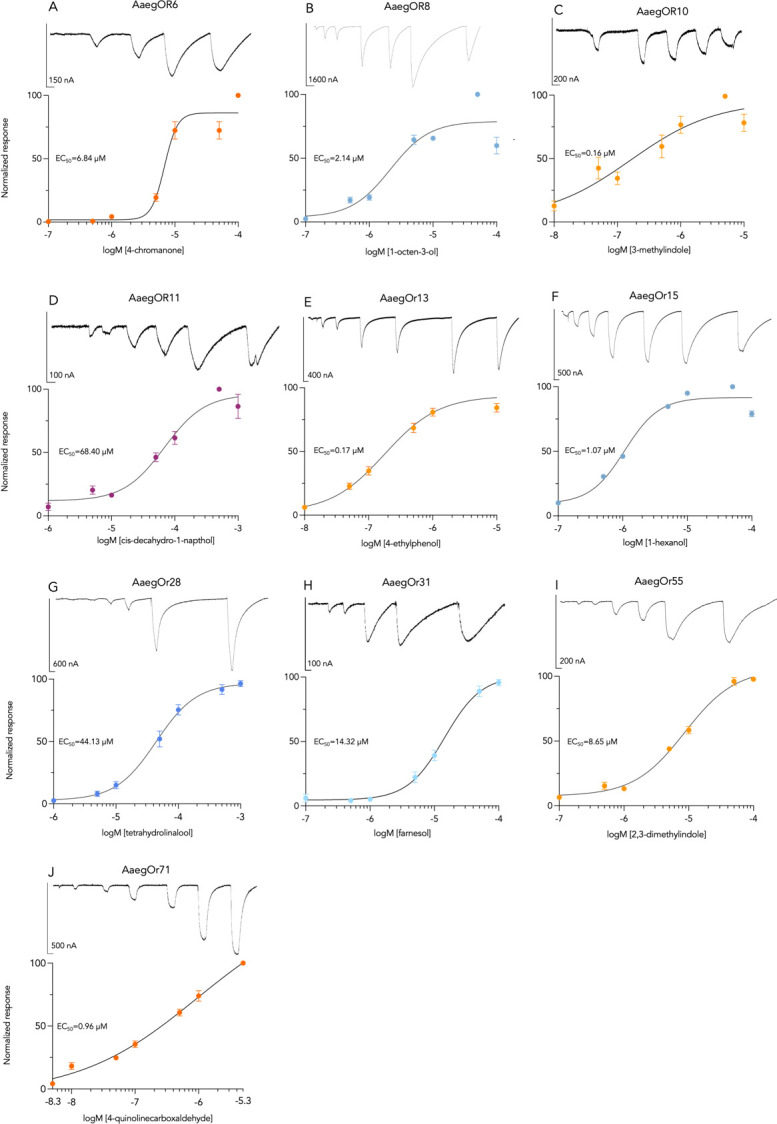
*Ae*. *aegypti* odorant receptor concentration-response curves. Normalized mean current responses of AaegORX/Orco expressing oocytes (n>10) to increasing log [M] concentrations of individual compounds. (A) 4-chromanone, (B) 1-octen-3-ol, (C) 3-methylindole (D) cis-decahydro-1-napthol, (E) 4-ethylphenol, (F) 1-hexanol, (G) tetrahydrolinalool, (H) farnesol, (I) 2,3-dimethylindole, and (J) 4-quinolinecarboxyaldehyde. Representative traces from individual oocyte recordings are shown above each curve, and EC_50_ values are given in figure insets.

Oocytes expressing *Ae*. *aegypti* ORs responded robustly to blends of chemical compounds (Fig 2 and S6 File). While we observed some low-level activation to multiple blends by each AaegOR (Fig 2), all demonstrated a pronounced selectivity for one blend over the others, as indicated by highly positive kurtosis values (Table 2). The blends that most frequently activated ORs were indole or alcohol blends. Unitary compounds from the highest activating blend for each OR were tested individually. We determined the primary ligand for each AaegOR from our chemical library and, in some cases, noted other secondary ligands (Table 2 and Fig 3). As with the blends, ORs exhibited pronounced activation by a single chemical compound, again supported by highly positive kurtosis values (Table 2). A defining feature of chemical receptor responsiveness is concentration-dependent activation. To investigate this aspect of OR function, we challenged oocytes with increasing concentrations of unitary compounds and estimated their potencies by calculating the half-maximal effective concentration (EC_50_) for each primary ligand (Table 2 and Fig 4).

**Table 2:** Functional responses of *Ae*. *aegypti* odorant receptors.

Odorant Receptor	Blends	Kurtosis	Primary Ligand	Kurtosis	EC50	Secondary Ligand
AaegOR6	Indoles 2	14.86	4-chromanone	10.85	6.84	
AaegOR8	Alcohols 2	15.71	1-octen-3-ol	8.75	2.14	
AaegOr10	Indoles 1	15.61	3-methylindole	5.87	0.16	
AaegOR11	Terpenes 3	5.68	cis-deca-hyfdro-1-naphol	6.85	68.4	fenchone
AaegOR13	Indoles 1	6.24	4-ethylphenol	9.11	0.17	
AaegOR15	Alcohols 2	3.63	1-hexanol	5.23	1.07	phenethyl propionate
AaegOR28	Alcohols 3	15.96	tetrahydrolinalool	7.7	44.13	
AaegOR31	Alcohols 1	14.58	farnesol	10.7	14.32	
AaegOR55	Indoles 1	14.50	2,3-dimethylindole	0.22	8.65	
AaegOR71	Indoles 2	15.56	4-quinolinecarboxaldehyde	3.08	0.96	

Summary of 10 *Ae*. *aegypti* odorant receptors and their corresponding responses to chemical blends and unitary compounds. Kurtosis values are provided for each blend and unitary compound. EC_50_ values in μM are indicated for primary ligands. Secondary ligands are presented if identified during the functional characterizations.

Three AaegORs responded robustly to the Indole 1 blend (Fig 2) with distinct unitary responses (Fig 3) producing potencies in the low micromolar range (Table 2 and Fig 4). For example, OR10, OR13, and OR55 responded to 3-methylindole (EC_50_ ∼0.16 μM), 4-ethylphenol (EC_50_ ∼0.17 μM), and 2,3-dimethylindole (EC_50_ ∼8.65 μM), respectively (Figs 3 and 4). OR6 and OR71 responded with the highest efficacies to the Indole 2 blend (Fig 2) and low-micromolar concentration-dependent responses to 4-chromanone (EC_50_ ∼6.84 μM) and 4-quinoline carboxaldehyde (EC_50_ ∼0.96 μM), respectively (Figs 3 and 4). Four AaegORs were activated by one or more alcohols from various blends (Fig 3): OR31 was specifically activated by farnesol, a sesquiterpene compound, from the Alcohols 1 blend (EC50 ∼14.32 μM), OR8 responded with the highest efficacy to 1-octen-3-ol (EC_50_ ∼2.14 μM), OR15 responded best to 1-hexanol (EC_50_ ∼1.07 μM) with additional responses to phenethyl propionate and other gree-leaf volatiles trans-2-hexanol and cis-3-henen-2-ol, and OR28 responded to tetrahydrolinalool (EC_50_ ∼44.13 μM) from the Alcohols 3 blend (Figs 3 and 4). Finally, the only receptor to respond to a compound not belonging to an indole or alcohol blend was OR11, which responded to cis-decahydro-1-naphthal (EC_50_ ∼68.40 μM) from the Terpenes 3 blend (Figs 3 and 4). Overall, we successfully identified ligands for a number of AaegORs that provide new insights into their potential function in physiological sensitivities of olfactory sensory neurons and, ultimately, behavioral responses to floral odors, as discussed below.

## Discussion

For mosquitoes, nectar foraging to obtain sugar meals is essential for survival. Moreover, individuals who locate and access higher-quality nectar sources exhibit extended lifespans and increased fecundity [47, 64, 65]. Therefore, exploring the molecular mechanisms driving selective preferences is imperative to a more complete understanding of mosquito chemical ecology. While prior studies have demonstrated the existence of nectar source preferences in various mosquito species corresponding to nectar qualities [49, 50, 66–70], a substantial gap in our comprehension of the sensory components facilitating these preferences remains. In other insect species, investigating OR function has been instrumental in deciphering behavioral patterns and formulating effective control measures [71–73].

ORs function as sensory gateways, providing critical information about the surrounding environment and enabling mosquitoes to discern and select nectar sources based on the chemical signals they receive [3]. In this context, our research centers on the responsiveness of a series of ORs to a library of ecologically relevant compounds, linking OR-ligand relationships to floral- or plant-derived odor sensing. By expanding our knowledge of the chemoreceptive basis of this critical mosquito sensory function, we expect to contribute to enhanced surveillance and control measures by developing more effective MBAKS. In turn, these improvements may help to mitigate the impact of mosquitoes on human health.

We initiated this study by examining *Ae*. *aegypti* OR gene structures, expression patterns, and relatedness to ORs from two other prominent disease vectors, *Ae*. *albopictus* and *Cx*. *quinquefasciatus* [43, 57, 61, 74]. While we have identified numerous chemoreceptors of interest in the three mosquito species that could respond to floral odors, we selected a subset of highly conserved ORs for our initial investigations in *Ae*. *aegypti* (Fig 1 and S3 File). Given their well-characterized response profiles, we selected AaegORs 8 and 10 as a means of verifying the accuracy of our heterologous cell expression platform [61, 74, 77]. Additional *Ae*. *aegypti* ORs were chosen because of their homologies to ORs that have been previously shown to respond to floral odors or other behaviorally relevant volatile compounds and the potential to identify novel ligands in our compound library (S6 File) [6, 43, 57, 75–78]. While AaegORs are expressed in various chemosensory tissues, antennae are the primary structures involved in odor sensing [1]. Their expression in the antennae of both sexes suggests that ORs are used for shared behaviors, such as nectar feeding (Table 1 and S5 File). Furthermore, by refining and examining *Ae*. *aegypti* ORs gene structures, we identified homologs of interest in both *Ae*. *albopictus* and *Cx*. *quinquefasciatus*, two medically important mosquito species. The existence of numerous closely related genes leads us to propose that the essential act of nectar foraging in vector mosquitoes shares a common underlying OR mechanism, indeed that OR-ligand pairs may have been conserved through the divergence of these species. Future studies will be required to characterize the response profiles of conserved ORs and to establish the requirement of these receptors in nectar foraging in *Ae*. *aegypti* and other species.

Using two-electrode voltage clamping in conjunction with the *Xenopus laevis* oocyte expression system, we assessed the responses of the AaegORs to our chemical library, which contains 147 compounds organized into 14 blends (S6 File). Our library was intentionally developed to include compounds that have been shown to influence insect behaviors. Many AaegORs responded robustly to the blends containing indole or indole-like compounds (Fig 2 and S7 File). Indoles are relatively ubiquitous in natural settings and are present in potential nectar sources [81, 82]. AaegOR71 responded to 4-quinoline carboxaldehyde, a compound identified in honey samples and endophytic bacteria, via gas chromatography [79, 80]. However, indoles are emitted naturally from many sources, including flowers, vertebrate hosts, and oviposition sites of microbiotic origin, suggesting they influence multiple behaviors across species [81, 82]. AaegOR6 responded best to 4-chromanone, a compound that is found in shrubs of the Asteraceae family, is structurally related to quinolines, and is a key constituent of flavonoid skeletons [83, 84]. AaegOR10 responded to 3-methylindole (skatole) found in animal feces and produces behavioral responses in other Dipteran species [85–88], while AaegOR55 responded to the structurally related compounds 2,3-dimethylindole and 3-methylindole. The best ligand identified for AaegOR13 was 4-ethylphenol, a volatile constituent of foral odors that is found in wine and has been linked to oviposition behavior in *Culex pipiens* [89, 90]. Other ORs were activated by plant-associated compounds from the alcohol blends. For example, AaegOR8 responded to 1-octen-3-ol, a well-characterized response to a floral odor and fungal volatile that variously attracts or deters insects depending on concentration [77, 91, 92]. A green-leaf volatile, 1-hexanol, is another compound with well-characterized physiological effects in many arthropods [93–96]. In our study, we discovered that 1-hexanol activates AaegOR15 with the highest efficacy and a very low threshold of response (EC_50_ ∼1.07 μM), as compared with phenethyl propionate identified in a previous study [43]. AaegOR28 responded best to tetrahydrolinalool, with lower responses to the isomers of linalool, which are plant/floral-derived compounds that have been shown to activate ORs from Anophelines [58]. AaegOR31 responds to farnesol, a common sesquiterpene extract in plant essential oils and a significant constituent of the honeybee Nasanov pheromone [97, 98]. Lastly, the AaegOR11 ligand, cis-decahydro-1-napthol, is structurally related to geosmin, a known oviposition attractant [99]. One limitation of oocyte studies is the number of compounds that can be screened. More efficacious or potent ligands may exist for AaegOR11 and AaegOR28, which resulted in estimated EC_50_ values greater than 40μM (Table 2). In the future, we will expand our library to further explore structure-activity relationships for ligand-receptor pairs identified in this study.

While ligands for four of the ORs in this study (AaegORs 8, 10, 11, and 15) had been identified in prior studies, we utilized them as positive controls and to search for new activating compounds, as our chemical library is more expansive than the ones previously tested. For AaegOR8 and AaegOR10, our results confirm high selectivity and sensitivity to 1-octen-3-ol and skatole, respectively [61, 100]. However, for AaegOR11 and AaegOR15, we discovered novel ligands with higher efficacies than those previously described. For AaegOR11, we found that cis-decahydro-1-napthol produced a greater magnitude of response than (-)-fenchone or (+)-fenchone [101], and we identified 1-hexanol as a potent activator of AaegOR15 in addition to phenethyl propionate [43]. This demonstrates that even for deorphanized ORs, assessing their function with expanded chemical libraries can aid in improving our understanding receptor-ligand interactions.

*Ae*. *aegypti* and other vector mosquitoes remain persistent global threats to human health and flourishing. Pursuing receptor-ligand pairings is paramount, as it opens doors to identifying potential attractants for behavioral control or targets for genetic knockout. In our investigation of floral-odor sensing receptors, we aim to pinpoint compounds that impart behavioral attraction for both sexes that can be applied in MBAKS. While these technologies have been successfully deployed in some studies [54–56], identifying compounds that activate *Ae*. *aegypti* ORs offer the potential to refine and enhance their effectiveness while minimizing the impact on non-target species. OR deorphanization is a crucial initial step in unraveling the intricate sensory systems of arthropods, providing valuable insights for future experiments and control strategies. While we have demonstrated OR- ligand interacts on a molecular level, more research is needed to determine how these ligands might influence behavior. Future studies will include olfactometry with wild-type and gene-knockout mosquitoes to verify the roles of these ORs and potential changes in their expression patterns in response to ligand exposure [102]. These studies can help to identify specific attractants in surveillance systems or MBAKS to monitor and control mosquito populations and combat the spread of disease. This focused endeavor not only aids in addressing the immediate threats posed by vector mosquitoes but also contributes to our broader understanding of insect behavior and the development of sustainable control measures.

## Supporting information

S1 File RNA sequencing validation of ORs. (DOCX)

S2 File Alignments of conceptual translations of AaegORs. (DOCX)

S3 File Odorant receptor homologs in *Aedes aegypti*, *Aedes albopictus*, and *Culex quinquefasciatus*. (XLSX)

S4 File Odorant receptor homolog alignments and intron positions. (DOCX)

S5 File Expression of *Aedes aegypti* odorant receptors in adult head appendages. (XLSX)

S6 File List of chemical compounds. (XLSX)

S7 File Raw data for two-electrode voltage clamping. (XLSX)
