# Supplementary material for: Odorant receptors for floral- and plant-derived volatiles in the yellow fever mosquito, Aedes aegypti (Diptera: Culicidae)
Source: PLoS One. 2024 May 6;19(5):e0302496. doi: 10.1371/journal.pone.0302496 (PMC11073699; doi:10.1371/journal.pone.0302496)

**RNA sequencing validation of ORs.**

Screen captures from VectorBase (www.vectorbase.org) showing gene structures of ten selected *Ae. aegypti* odorant receptors (*AaegOr*) with compiled unique RNAseq reads (orange bars) derived from non-bloodfed female antennae and maxillary palps (Matthews *et al*., 2016). The scale on the left indicates the number of reads. Red and blue boxes represent exons. Numbers at the top of each image refer to base pair positions within the AaegL5 assembly. VectorBase gene ID and descriptions are shown in black and blue text, respectively.

*AaegOr6* – female antennae


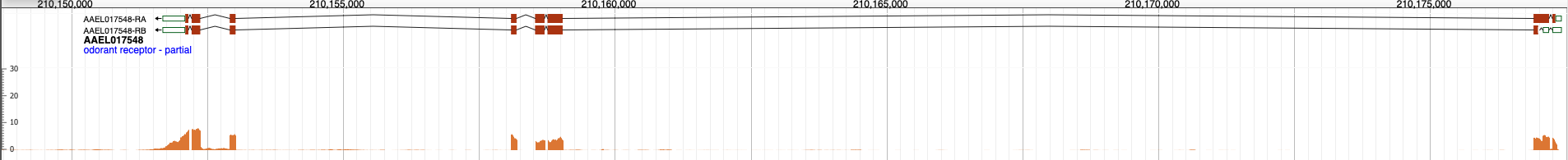


*AaegOr8* – female maxillary palps


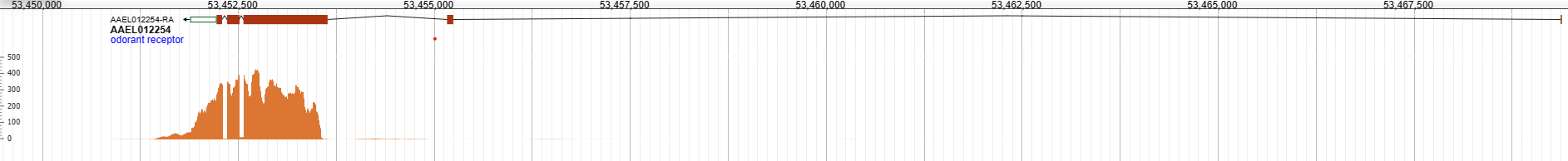


*AaegOr10* – female antennae


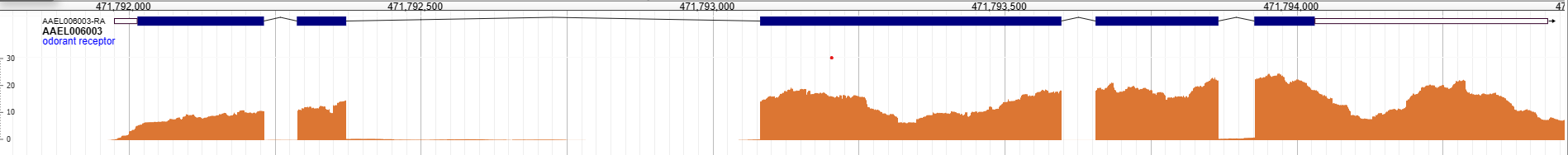


*AaegOr11* – female antennae


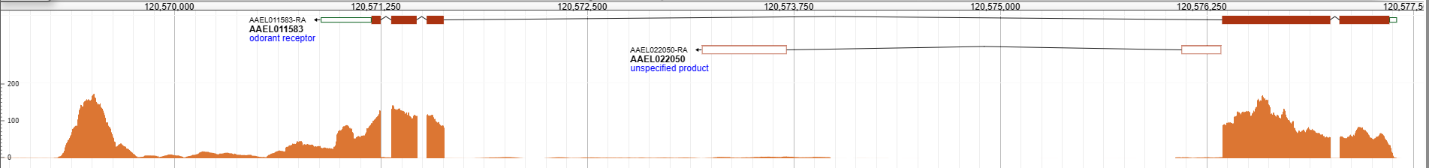


*AaegOr13* – female antennae


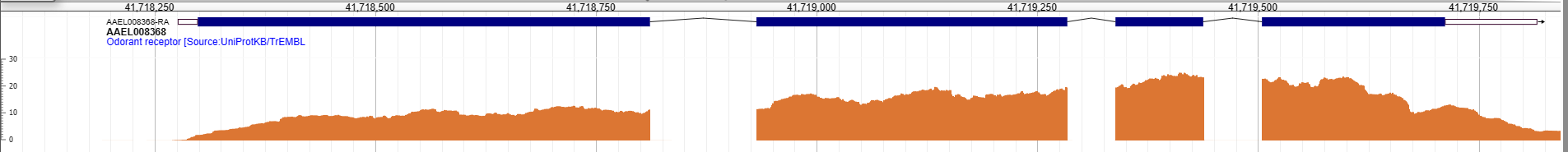


*AaegOr15* – female antennae


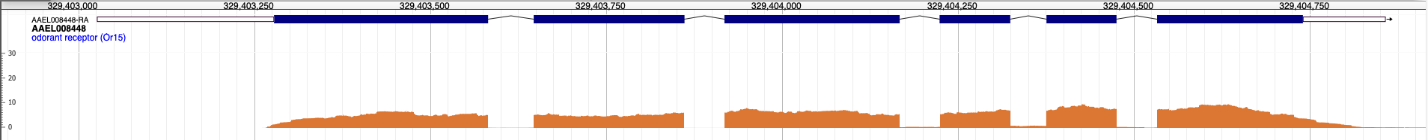


*AaegOr28* – female antennae


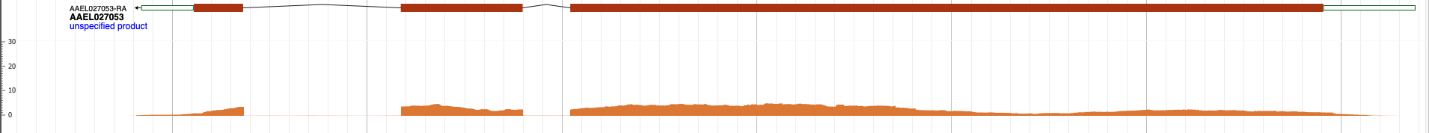


*AaegOr31* – female antennae


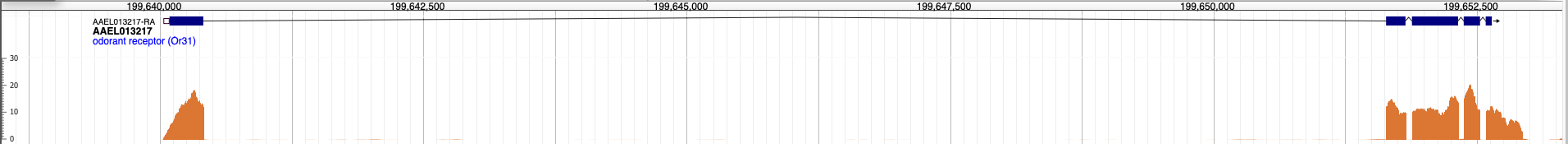


*AaegOr55* – female antennae


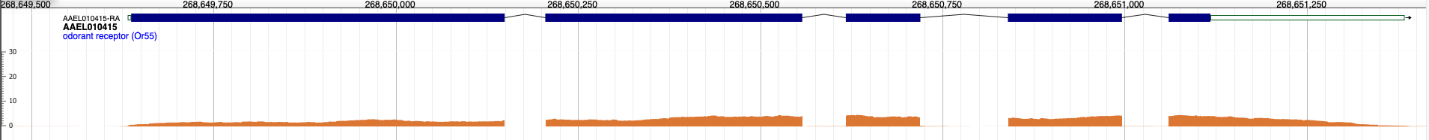


*AaegOr71* – female antennae


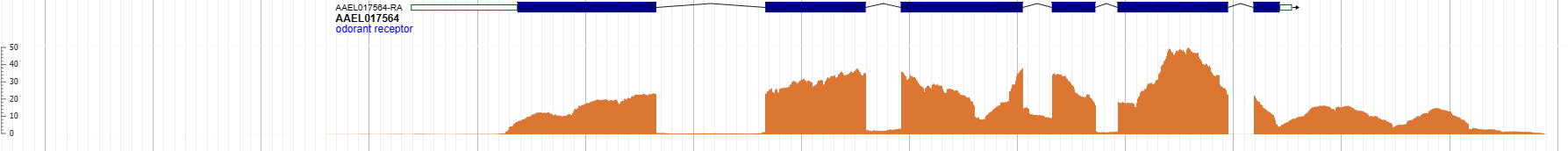

Supplement: S1 File — (DOCX) [file pone.0302496.s001.docx]
