# Supplementary material for: Odorant receptors for floral- and plant-derived volatiles in the yellow fever mosquito, Aedes aegypti (Diptera: Culicidae)
Source: PLoS One. 2024 May 6;19(5):e0302496. doi: 10.1371/journal.pone.0302496 (PMC11073699; doi:10.1371/journal.pone.0302496)
Supplement: S2 File — (DOCX) [file pone.0302496.s002.docx]

**Alignments of conceptual translations of AaegORs**

*De novo* transcripts were generated with the Trinity software package [42] in Galaxy (usegalaxy.org) using *Aedes aegypti* non-bloodfed female antennae or maxillary palp RNAseq reads [41, 63]. Conceptual translations from the *de novo* assemblies of the ten AaegORs in this study were aligned with peptide sequences from the most recent AaegL5 genome assembly. Sequences were aligned using ClustalW with character counts [64]. VectorBase IDs of the AaegL5 transcripts and Trinity *de novo* transcript names are provided on the left. Identical residues are highlighted in gray and divergent residues are shown in **bold type**. End line numbers refer to amino acid positions.

**AaegOR6**

AaegOr6_AAEL017548-RA MVSFEQSFTAVDLILITSGIPSCSSFYPVSIKSALKRNAVFLMAFTLLFYTAFGELVYLV 60

AaegOr6_Antenna_TRINITY_DN29927_c0_g2_i1 ------------------------------------------------------------ 0

AaegOr6_AAEL017548-RA EMLQRDFSFLEITFQAPCLGYCTIGLMKMLILAVKRNTIAELVQSLQEEWNKSVRSLEHQ 120

AaegOr6_Antenna_TRINITY_DN29927_c0_g2_i1 ------------------------------------------------------------ 0

AaegOr6_AAEL017548-RA SICDDVMKPAIRFTTIVAVVNIVMGLAFTLLPIPEMIYYYSTHGKWVRQLPFLIWWSFDA 180

AaegOr6_Antenna_TRINITY_DN29927_c0_g2_i1 ------------------------------------------------------------ 0

AaegOr6_AAEL017548-RA YSGFVYYFIYPLYVVIGFSGIIIHMGFDCLFCILSAHLCVHLRILKFDLENLTYGLDSSE 240

AaegOr6_Antenna_TRINITY_DN29927_c0_g2_i1 ------------------------------------------------LENLTYGLDSSE 12

AaegOr6_AAEL017548-RA NMSIKLNPKLFYIVEKHQNILECHDRMNKIFNFALFYNFFVSSFIICIQGFMVTAASGYT 300

AaegOr6_Antenna_TRINITY_DN29927_c0_g2_i1 NMSIKLNPKLFYIVEKHQNILECHDRMNKIFNFALFYNFFVSSFIICIQGFMVTAASGYT 72

AaegOr6_AAEL017548-RA LIKFALFLASFLVELFLLCFYGHHIVESSVLVAEAAYNCLWYNTNHQFRTIILQMVNKGQ 360

AaegOr6_Antenna_TRINITY_DN29927_c0_g2_i1 LIKFALFLASFLVELFLLCFYGHHIVESSVLVAEAAYNCLWYNTNHQFRTIILQMVNKGQ 132

AaegOr6_AAEL017548-RA IPLSLMAWKIWPVNMNTFANILSASWSYFTLIRTVYAD 398

AaegOr6_Antenna_TRINITY_DN29927_c0_g2_i1 IPLSLMAWKIWPVNMNTFANILSASWSYFTLIRTVYAD 170

**AaegOR8**

AaegOr8 MNDLVKFESFIRVPEIFFDMIGITRYGEARDTWKARLKQAFFWSSYANTIFCLIIEHIYF 60

AaegOr8_palp_TRINITY_DN10600_c0_g1_i2 MNDLVKFESFIRVPEIFFDMIGITRYGEARDTWKARLKQAFFWSSYANTIFCLIIEHIYF 60

AaegOr8 IKAAGNFTNFLELTALAPCIGFTALSIVKIMTIKLNEAKLNGILDRLSDLFPRSHLDQDR 120

AaegOr8_palp_TRINITY_DN10600_c0_g1_i2 IKAAGNFTNFLELTALAPCIGFTALSIVKIMTIKLNEAKLNGILDRLSDLFPRSHLDQDR 120

AaegOr8 YRTYNYNLESQMVMKSFSILYMILIWIFNLLPLVSMLVNYISTGILEKELPYFMWYWYDW 180

AaegOr8_palp_TRINITY_DN10600_c0_g1_i2 YRTYNYNLESQMVMKSFSILYMILIWIFNLLPLVSMLVNYISTGILEKELPYFMWYWYDW 180

AaegOr8 HKAGYYEITFFHQNWGAFDSAVFNLSTDLLFCAIILLICLQFDILAYRLRHAKGDYKELE 240

AaegOr8_palp_TRINITY_DN10600_c0_g1_i2 HKAGYYEITFFHQNWGAFDSAVFNLSTDLLFCAIILLICLQFDILAYRLRHAKGDYKELE 240

AaegOr8 QCVKLHQSVVELSNQLEGIFSPSILVNFVGSSVIICLVGFQATSNISAFDLFKFILFLIS 300

AaegOr8_palp_TRINITY_DN10600_c0_g1_i2 QCVKLHQSVVELSNQLEGIFSPSILVNFVGSSVIICLVGFQATSNISAFDLFKFILFLIS 300

AaegOr8 SLVQVFLLCYYGNKLIEASSQIGYCAFEGTWYMADLRYQKSLLFVMTRAGQWQKLTAMKF 360

AaegOr8_palp_TRINITY_DN10600_c0_g1_i2 SLVQVFLLCYYGNKLIEASSQIGYCAFEGTWYMADLRYQKSLLFVMTRAGQWQKLTAMKF 360

AaegOr8 SVVSLASYSAILSTSFSYFTLLKTIYEPSQK 391

AaegOr8_palp_TRINITY_DN10600_c0_g1_i2 SVVSLASYSAILSTSFSYFTLLKTIYEPSQK 391

**Aaeg0R10**

AaegOr10_AAEL006003-RA MASILDCPIVSVNARVWRFWSFVLKHDAMRYISIIPVTVMTFFMFTDLCRSWGNIQELII 60

AaegOr10_Antenna_TRINITY_DN1780_c0_g1_i10 MASILDCPIVSVNARVWRFWSFVLKHDAMRYISIIPVTVMTFFMFTDLCRSWGNIQELII 60

AaegOr10_AAEL006003-RA KAYFAVLYFNAVLRTLILVKDRKLYENFMQGISNVYFEISHIDDHKIQSLLKSYTVRARM 120

AaegOr10_Antenna_TRINITY_DN1780_c0_g1_i10 KAYFAVLYFNAVLRTLILVKDRKLYENFMQGISNVYFEISHIDDHKIQSLLKSYTVRARM 120

AaegOr10_AAEL006003-RA LSISNLALGAIISTCFVVYPIFTGERGLPYGMFIPGLDSFRSPHYEIIYIVQVVLTFPGC 180

AaegOr10_Antenna_TRINITY_DN1780_c0_g1_i10 LSISNLALGAIISTCFVVYPIFTGERGLPYGMFIPGLDSFRSPHYEIIYIVQVVLTFPGC 180

AaegOr10_AAEL006003-RA CMYIPFTSFFASTTLFGLVQIKTLQRQLQTFKDNI**S**SQDKEKVK**T**KVVKLIEDHKRIITY 240

AaegOr10_Antenna_TRINITY_DN1780_c0_g1_i10 CMYIPFTSFFASTTLFGLVQIKTLQRQLQTFKDNI**N**SQDKEKVK**A**KVVKLIEDHKRIITY 240

AaegOr10_AAEL006003-RA VSELNSLVTYICFVEFLSFGMMLCALLFLLNVIENHAQIVIVAAYIFMIISQIFAFYWHA 300

AaegOr10_Antenna_TRINITY_DN1780_c0_g1_i10 VSELNSLVTYICFVEFLSFGMMLCALLFLLNVIENHAQIVIVAAYIFMIISQIFAFYWHA 300

AaegOr10_AAEL006003-RA NEVREESMNLAEAAYSGPWVELDNSIKKKLLLIILRAQQPLEITVGNVYPMTLEMFQSLL 360

AaegOr10_Antenna_TRINITY_DN1780_c0_g1_i10 NEVREESMNLAEAAYSGPWVELDNSIKKKLLLIILRAQQPLEITVGNVYPMTLEMFQSLL 360

AaegOr10_AAEL006003-RA NASYSYFTLLRRVYN 375

AaegOr10_Antenna_TRINITY_DN1780_c0_g1_i10 NASYSYFTLLRRVYN 375

**AaegOR11**

AaegOr11_AAEL011583-RA MQLKDEWIQEDDVYDNPLLRLTINGLKYYGILLYKSQPFKKLNCFRGVCFTASMLAFNVT 60

AaegOr11_Antenna_TRINITY_DN1275_c0_g1_i2 MQLKDEWIQEDDVYDNPLLRLTINGLKYYGILLYKSQPFKKLNCFRGVCFTASMLAFNVT 60

AaegOr11_AAEL011583-RA QYVDLYQVWGNIAEMTANAATTLLFTTTIVRILHFYWNRARFNNAIKVADEGVQHLLRFG 120

AaegOr11_Antenna_TRINITY_DN1275_c0_g1_i2 QYVDLYQVWGNIAEMTANAATTLLFTTTIVRILHFYWNRARFNNAIKVADEGVQHLLRFG 120

AaegOr11_AAEL011583-RA NAPEKEIFWDNVKYMNRLTAAFWICALVTANTMCVYALVQYQ**S**LKS**M**DSFNSTEPFDPPT 180

AaegOr11_Antenna_TRINITY_DN1275_c0_g1_i2 NAPEKEIFWDNVKYMNRLTAAFWICALVTANTMCVYALVQYQ**T**LKS**I**DSFNSTEPFDPPT 180

AaegOr11_AAEL011583-RA ILRSWYPTDNIVDSFATIYLIQLYIMYVGQLIVPCWHVFMVSLMLYARTALMALNYKLA**N** 240

AaegOr11_Antenna_TRINITY_DN1275_c0_g1_i2 ILRSWYPTDNIVDSFATIYLIQLYIMYVGQLIVPCWHVFMVSLMLYARTALMALNYKLA**H** 240

AaegOr11_AAEL011583-RA LEQYAVSG**M**RSGRKIKCVVDPEEERCNVRKELIVECIQQQHKIFEYTRELEALTRGAMFM 300

AaegOr11_Antenna_TRINITY_DN1275_c0_g1_i2 LEQYAVSG**I**RSGRKIKCVVDPEEERCNVRKELIVECIQQQHKIFEYTRELEALTRGAMFM 300

AaegOr11_AAEL011583-RA DFVVFSVLLCALLFEASSTNSFVQIFIDICYIMTMTAMLFLYYWHANEIHYQANLLSSSA 360

AaegOr11_Antenna_TRINITY_DN1275_c0_g1_i2 DFVVFSVLLCALLFEASSTNSFVQIFIDICYIMTMTAILFLYYWHANEIHYQANLLSSSA 360

AaegOr11_AAEL011583-RA FMNDWYNYPRSVNRHLITFICYSNKPLDMKAYIVSMSLDTFLAILRASYSYFTILKQAAG 420

AaegOr11_Antenna_TRINITY_DN1275_c0_g1_i2 FMNDWYNYPRSVNRHLITFICYSNKPLDMKAYIVSMSLDTFLAILRASYSYFTILKQAAG 420

**AaegOR13**

AaegOr13_AAEL008368-RA MWQPLRKFLAPGPELLSFGLQMLRFIGLWGDRRQVVRYLLVLFSEFIFLIGPKALLGSDK 60

AaegOr13_Antenna_TRINITY_DN9791_c2_g1_i2 MWQPLRKFLAPGPELLSFGLQMLRFIGLWGDRRQVVRYLLVLFSEFIFLIGPKALLGSDK 60

AaegOr13_AAEL008368-RA EGFDSTARNIGELIFLVEVCISIGIFASRRASFERLIVVLENILRRKWPRNLQDEIYRFH 120

AaegOr13_Antenna_TRINITY_DN9791_c2_g1_i2 EGFDSTARNIGELIFLVEVCISIGIFASRRASFERLIVVLENILRRKWPRNLQDEIYRFH 120

AaegOr13_AAEL008368-RA RRMEFFARAYALYIGFLLFLYNCVPIGSTIVKLIRFDESERSDFMLVVELQFFWFDIRRN 180

AaegOr13_Antenna_TRINITY_DN9791_c2_g1_i2 RRMEFFARAYALYIGFLLFLYNCVPIGSTIVKLIRFDESERSDFMLVVELQFFWFDIRRN 180

AaegOr13_AAEL008368-RA VVHYAIYMAFCFVAVSCSAYQSTLKGSVIVVVTQYGSKLFELISKRIDAMRSIERAADRD 240

AaegOr13_Antenna_TRINITY_DN9791_c2_g1_i2 VVHYAIYMAFCFVAVSCSAYQSTLKGSVIVVVTQYGSKLFELISKRIDAMRSIERAADRD 240

AaegOr13_AAEL008368-RA RELREIVKLHGMALEYVQHLESTISFVMINQIMNCIFIWCLMMFYVSTNFGPNAANVMLL 300

AaegOr13_Antenna_TRINITY_DN9791_c2_g1_i2 RELREIVKLHGMALEYVQHLESTISFVMINQIMNCIFIWCLMMFYVSTNFGPNAANVMLL 300

AaegOr13_AAEL008368-RA FLVLMGEMVVYCLNGTALSEQAAGVGHAIYNYPWYKESVAMQKNMQLMIQRAQRPTGITA 360

AaegOr13_Antenna_TRINITY_DN9791_c2_g1_i2 FLVLMGEMVVYCLNGTALSEQAAGVGHAIYNYPWYKESVAMQKNMQLMIQRAQRPTGITA 360

AaegOr13_AAEL008368-RA AKFYFVNIERLGLVTQASYSYYLILKNRF 389

AaegOr13_Antenna_TRINITY_DN9791_c2_g1_i2 AKFYFVNIERLGLVTQASYSYYLILKNRF 389

**AaegOR15**

AaegOr15_AAEL008448-RA MK**Y**FELTEPEAAMPLALRLLETYGLRGGKRKFLQFQVTILWELLMIVIPKIVFGYRSQDL 60

AaegOr15_Antenna_TRINITY_DN448_c0_g1_i4 --**H**FELTEPEAAMPLALRLLETYGLRGGKRKFLQFQVTILWELLMIVIPKIVFGYRSQDL 58

AaegOr15_AAEL008448-RA VIRGLSELLFQLHIMIRISIFAWHRFKYESLIDIIRKVYRKTFSTGGDPTSKSIILKFNQ 120

AaegOr15_Antenna_TRINITY_DN448_c0_g1_i4 VIRGLSELLFQLHIMIRISIFAWHRFKYESLIDIIRKVYRKTFSTGGDPTSKSIILKFNQ 118

AaegOr15_AAEL008448-RA MINKQSKGYFLYIMGCVSLFSVAPVVQSVIIFMANQSRNGTEKAEYVTMMEQEFYGLDIR 180

AaegOr15_Antenna_TRINITY_DN448_c0_g1_i4 MINKQSKGYFLYIMGCVSLFSVAPVVQSVIIFMANQSRNGTEKAEYVTMMEQEFYGLDIR 178

AaegOr15_AAEL008448-RA GNFGHYAIYVALAGLAHYYSASFFAVTGVIIICGVRCTILTFKLINVRLSKLHELPKQDI 240

AaegOr15_Antenna_TRINITY_DN448_c0_g1_i4 GNFGHYAIYVALAGLAHYYSASFFAVTGVIIICGVRCTILTFKLINVRLSKLHELPKQDI 238

AaegOr15_AAEL008448-RA RDELREIIDLHVDALRCIQLLEQIANLAMVIQIIDCVLIWISMILY**M**RNNLGVDAISLMV 300

AaegOr15_Antenna_TRINITY_DN448_c0_g1_i4 RDELREIIDLHVDALRCIQLLEQIANLAMVIQIIDCVLIWISMILY**I**RNNLGVDAISLMV 298

AaegOr15_AAEL008448-RA LFVALTGETYALCDLLTQLTSESLAVTRAIIDCQWYSLPLDVQKSLSFVLFRAQRKEGIT 360

AaegOr15_Antenna_TRINITY_DN448_c0_g1_i4 LFVALTGETYALCDLLTQLTSESLAVTRAIIDCQWYSLPLDVQKSLSFVLFRAQRKEGIT 358

AaegOr15_AAEL008448-RA AAKFFFMDIERFGSVAQTSYSIYVVLKDQL 390

AaegOr15_Antenna_TRINITY_DN448_c0_g1_i4 AAKFFFMDIERFGSVAQTSYSIYVVLKDQL 388

**AaegOR28**

AaegOr28_AAEL027053-RA MVWIEADRSSSLEYDSFFRLPKIFGLLNGVVYNDEKPSSKWSKAKNVYFWISLMHSILVA 60

AaegOr28_Antenna_TRINITY_DN41586_c0_g1_i1 MVWIEADRSSSLEYDSFFRLPKIFGLLNGVVYNDEKPSSKWSKAKNVYFWISLMHSILVA 60

AaegOr28_AAEL027053-RA VLELVYLAKSVEQNADFVFIMSLVPLVGHGILAIVKLSVQKYYHKEINSILISLKGIYPS 120

AaegOrO28_Antenna_TRINITY_DN41586_c0_g1_i1 VLELVYLAKSVEQNADFVFIMSLVPLVGHGILAIVKLSVQKYYHKEINSILISLKGIYPS 120

AaegOr28_AAEL027053-RA TLDDTITKDYSKKILYMKLFVIFYLVTLIFFNIVPFAPVLHTYFTTGVFEKTLPFFIYYW 180

AaegOr28_Antenna_TRINITY_DN41586_c0_g1_i1 TLDDTITKDYSKKILYMKLFVIFYLVTLIFFNIVPFAPVLHTYFTTGVFEKTLPFFIYYW 180

AaegOr28_AAEL027053-RA YDWRRPILYELTFIEQIWVSTASVVANMNIDLMLCSLILQISMHFDVLSDRLSVLQHNDH 240

AaegOr28_Antenna_TRINITY_DN41586_c0_g1_i1 YDWRRPILYELTFIEQIWVSTASVVANMNIDLMLCSLILQISMHFDVLSDRLSVLQHNDH 240

AaegOr28_AAEL027053-RA KELAKCVERHSVLLDLCLRVENIFSRSMLASFLLSSVIICLTGFQVFAQDSINKAIPYAT 300

AaegOr28_Antenna_TRINITY_DN41586_c0_g1_i1 KELAKCVERHSVLLDLCLRVENIFSRSMLASFLLSSVIICLTGFQVFAQDSINKAIPYAT 300

AaegOr28_AAEL027053-RA FLFLHMVDVYLLCYYGNLMMEKSL**A**VSNYAYESLWYLGNRPFQKSILIILERGQRAQ**K**LT 360

AaegOr28_Antenna_TRINITY_DN41586_c0_g1_i1 FLFLHMVDVYLLCYYGNLMMEKSL**D**VSNYAYESLWYLGNRPFQKSILIILERGQRAQ**E**LT 360

AaegOr28_AAEL027053-RA AMKFI**D**INLTCFKTILSTSFSYFTLLKVLNEPME 394

AaegOr28_Antenna_TRINITY_DN41586_c0_g1_i1 AMKFI**V**INLTCFKTILSTSFSYFTLLKVLNEPME 394

**AaegOR31**

AaegOr31_AAEL013217-RA MAPTQNGRDREKFLRVQLLCLALIGIKRHETVSSRTIFHVCFISMVIMDLATILFALEHA 60

AaegOr31_Antenna_TRINITY_DN6991_c0_g1_i1 MAPTQNGRDREKFLRVQLLCLALIGIKRHETVSSRTIFHVCFISMVIMDLATILFALEHA 60

AaegOr31_AAEL013217-RA NDIALVCDCLGPTFTAYLGIVKQYCLSAHRVELWNIIETLRRLKDYAG**I**SEIESIERNNK 120

AaegOr31_Antenna_TRINITY_DN6991_c0_g1_i1 NDIALVCDCLGPTFTAYLGIVKQYCLSAHRVELWNIIETLRRLKDYAG**T**SEIESIERNNK 120

AaegOr31_AAEL013217-RA IDRFLATAYLMSASATGSLFIIAALAKGCYKLIFQNIIEWGFPLSLSFPFKTSHPIVFG**M** 180

AaegOr31_Antenna_TRINITY_DN6991_c0_g1_i1 IDRFLATAYLMSASATGSLFIIAALAKGCYKLIFQNIIEWGFPLSLSFPFKTSHPIVFG**V** 180

AaegOr31_AAEL013217-RA FFVWSSAAIYIVVFCSVSSDASFGGLASNVVVHFKLLQKRLQDATFADNDENLKQLIEYH 240

AaegOr31_Antenna_TRINITY_DN6991_c0_g1_i1 FFVWSSAAIYIVVFCSVSSDASFGGLASNVVVHFKLLQKRLQDATFADNDENLKQLIEYH 240

AaegOr31_AAEL013217-RA SLLLNLSRKIMSSFRVIIINNLLVASVLLCVLGFQLVMFLGSTLMLIYLMYVTAIVIQIT 300

AaegOr31_Antenna_TRINITY_DN6991_c0_g1_i1 SLLLNLSRKIMSSFRVIIINNLLVASVLLCVLGFQLVMFLGSTLMLIYLMYVTAIVIQIT 300

AaegOr31_AAEL013217-RA FFAYYGSLL**L**HESEEVS**I**SIYCSNWYEASPKTRRILLQCLMRAQVPVNTKAGFMVASLPT 360

AaegOr31_Antenna_TRINITY_DN6991_c0_g1_i1 FFAYYGSLL**S**HESEEVS**S**SIYCSNWYEASPKTRRILLQCLMRAQVPVNTKAGFMVASLPT 360

AaegOr31_AAEL013217-RA LRAILNSAGSYVALLLSFTDN 381

AaegOr31_Antenna_TRINITY_DN6991_c0_g1_i1 LRAILNSAGSYVALLLSFTDN 381

**AaegOR55**

AaegOr55_AAEL010415-RA M**QQK**PPFRLMSASLKLC**R**WLGLWHD**V**NLDKPCWQTVF**ISF**CLLFWYILPG**CL**YI**T**RGE**R**M 60

AaegOr55_Antenna_TRINITY_DN13286_c0_g1_i6 -**NSQ**PPFRLMSASLKLC**K**WLGLWHD**A**NLDKPCWQTVF**LIL**CLLFWYILPG**YM**YI**V**RGE**K**M 59

AaegOr55_AAEL010415-RA LQ**Y**LLK**S**ILEVFSM**C**VIVLRC**VV**HMINR**KT**VQ**N**CF**VE**LQ**D**AIS**T**F**E**NSPYEDV**RQM**LRHL 120

AaegOr55_Antenna_TRINITY_DN13286_c0_g1_i6 LQ**D**LLK**P**ILEVFSM**A**VIVLRC**LI**HMINR**QS**VQ**E**CF**AD**LQ**N**AIS**K**F**K**NSPYEDV**QKI**LRHL 119

AaegOr55_AAEL010415-RA LKSADY**L**VK**I**YVSIVF**I**QA**SI**YG**LVP**A**I**LTT**YR**YC**N**SNE**T**IQLPSAVM**E**ADYVLFDH**ST**N 180

AaegOr55_Antenna_TRINITY_DN13286_c0_g1_i6 LKSADY**I**VK**F**YVSIVF**V**QA**GL**YG**FLS**A**A**LTT**FK**YC**T**SNE**I**IQLPSAVM**D**ADYVLFDH**TV**N 179

AaegOr55_AAEL010415-RA YWIWL**L**VTI**I**SL**LV**EYL**L**LG**TF**S**S**QECLFWNLLHH**V**SSL**L**K**V**V**R**LEIARLDQY**T**DPKQ**YT** 240

AaegOr55_Antenna_TRINITY_DN13286_c0_g1_i6 YWIWL**P**VTI**V**SL**II**EYL**M**LG**SI**S**A**QECLFWNLLHH**I**SSL**F**K**I**V**H**LEIARLDQY**K**DPKQ**FK** 239

AaegOr55_AAEL010415-RA ERLA**S**IVS**T**HEVC**Y**R**C**AR**SL**E**I**VLSPLLA**VL**YC**T**CI**T**QTCYLLF**V**ISM**ID**D**L**VV**I**ASMIF 300

AaegOr55_Antenna_TRINITY_DN13286_c0_g1_i6 ERLA**F**IVS**I**HEVC**F**R**S**AR**CM**E**K**VLSPLLA**LW**YC**A**CI**A**QTCYLLF**F**ISM**VN**D**V**VV**V**ASMIF 299

AaegOr55_AAEL010415-RA VLQY**I**VFLIFSFSMLGAEL**T**EESA**L**VS**E**A**I**YNSNWYMRMPAERRLLLFMKMRADRPVGIT 360

AaegOr55_Antenna_TRINITY_DN13286_c0_g1_i6 VLQY**V**VFLIFSFSMLGAEL**M**EESA**R**VS**D**A**V**YNSNWYMRMPAERRLLLFMKMRADRPVGIT 359

AaegOr55_AAEL010415-RA AAKFFYVNRSTFAEAMKTAFSFFTIMQQFYGE 392

AaegOr55_Antenna_TRINITY_DN13286_c0_g1_i6 AAKFFYVNRSTFAEAMKTAFSFFTIMQ----- 386

**AaegOR71**

AaegOr71_AAEL017564-RA MELSYHRSLPPELQIMPFQLRCMELIGLIGPKGRFYRFVLAFGWGTFVILLPKSVLGIGS 60

AaegOr71_Antenna_TRINITY_DN13495_c0_g1_i23 MELSYHRSLPPELQIMPFQLRCMELIGLIGPKGRFYRFVLAFGWGTFVILLPKSVLGIGS 60

AaegOr71_AAEL017564-RA SELDAIIKGFAELLFEGNLFIAVASLVPKLPLVKRLLHVLSEIFRQATHDK**H**D**A**KDHCYA 120

AaegOr71_Antenna_TRINITY_DN13495_c0_g1_i23 SELDAIIKGFAELLFEGNLFIAVASLVPKLPLVKRLLHVLSEIFRQATHDK**R**D**V**KDHCYA 120

AaegOr71_AAEL017564-RA LICEQNSKIDKFCKFYFIYCCFGPFVFCIPAMVTSYVRYF**D**TTNNGTNANGSEHLRFELP 180

AaegOr71_Antenna_TRINITY_DN13495_c0_g1_i23 LICEQNSKIDKFCKFYFIYCCFGPFVFCIPAMVTSYVRYF**G**TTNNGTNANGSEHLRFELP 180

AaegOr71_AAEL017564-RA MEQEFYWLPIRTNFACYHLFLALSLSAYCVCSYMSVIKVSTLLIMIKYCSLVYRLVAIRI 240

AaegOr71_Antenna_TRINITY_DN13495_c0_g1_i23 MEQEFYWLPIRTNFACYHLFLALSLSAYCVCSYMSVIKVSTLLIMIKYCSLVYRLVAIRI 240

AaegOr71_AAEL017564-RA RELGKXPPGRKQDDTDEDERTKMVMVKEVVEMHEKALEATDLVEKVINIPIAMQFMACIL 300

AaegOr71_Antenna_TRINITY_DN13495_c0_g1_i23 RELGK*PPGRKQDDTDEDERTKMVMVKEVVEMHEKALEATDLVEKVINIPIAMQFMACIL 299

AaegOr71_AAEL017564-RA FWCMTMVYVSTNINFNLFNVMVLFWLSLIETYGYSYLGTELSEDAKAVGHAVYDLPWYED 360

AaegOr71_Antenna_TRINITY_DN13495_c0_g1_i23 FWCMTMVYVSTNINFNLFNVMVLFWLSLIETYGYSYLGTELSEDAKAVGHAVYDLPWYED 359

AaegOr71_AAEL017564-RA SAQLQRYYRLMIQRSQQNIGVTAAKFFIVGIEKFGKVVNLSYSYYLVLKDVLDRL 415

AaegOr71_Antenna_TRINITY_DN13495_c0_g1_i23 SAQLQRYYRLMIQRSQQNIGVTAAKFFIVGIEKFGKVVNLSYSYYLVLKDVLDRL 414
