# Supplementary material for: Odorant receptors for floral- and plant-derived volatiles in the yellow fever mosquito, Aedes aegypti (Diptera: Culicidae)
Source: PLoS One. 2024 May 6;19(5):e0302496. doi: 10.1371/journal.pone.0302496 (PMC11073699; doi:10.1371/journal.pone.0302496)
Supplement: S4 File — (DOCX) [file pone.0302496.s004.docx]

**Odorant receptor homolog alignments and intron positions**.

Amino acid alignments of *Aedes aegypti, Aedes albopictus,* and *Culex quinquefasciatus* with intron positions indicated in green. Intron phases, o, 1, and 2, are noted above. Gray shading indicates identical amino acids. Boldface type indicates conserved amino acids.

**OR6**

(1)

CQUJHB000029_OR6 M**YV**FE**D**T**LSSLN**L**MF**-**FAS**IP**P**C**NE**FYP**ET**I**RMIF**K**E**N**KF**FLIAF**S**LLLYT**QL**GEIVYL**A** 59

AAEL017548_OR6 MV**S**FEQ**S**F**T**AVDLILITSGIPSCSSFYP**V**SIKSALKRNAVFL**M**AFTLL**F**YTAFGELVYLV 60

AALFPA_042417_OR6 MV**K**FEQTF**K**AVDLILITSGIPSCSSFYP**A**SIKSALKRNAVFLIAFTLLLYTAFGELVYLV 60

* **:::.:::*:: :.** *..*** :*: :*.* .**:**:**:** :**:***.

(0)

CQUJHB000029_OR6 QM**V**QRE**C**SFLE**L**TFQAPC**V**GYCSIGL**L**KM**VVI**AVKRN**V**IAELVQ**N**L**RDV**W**DQ**S**IITV**EH**R** 119

AAEL017548_OR6 **E**MLQR**D**FSFLEITFQAPCLGYC**T**IGLMKMLILAVKRN**T**IAELVQ**S**LQ**EE**W**NK**SVRS**L**EHQ 120

AALFPA_042417_OR6 QMLQREF**T**FLEITFQAPCLGYCSIGLMKMLILA**M**KRN**S**IA**G**LVQ**T**LQ**KY**W**YT**SVRS**I**EHQ 120

:*:**: :***:******:***:***:**:::*:*** ** ***.*:. * *: ::**:

CQUJHB000029_OR6 **E**IC**KN**VMKPAI**SI**T**SLT**AVVN**VI**MG**IS**FTILPI**A**EMIYHY**ALA**G**G**WVRQLPFLI**C**W**P**FD**P** 179

AAEL017548_OR6 SICD**D**VMKPAIRFTTIVAVVNIVMG**L**AFTLLPIPEMIY**Y**Y**STH**G**K**WVRQLPFLIWWSFD**A** 180

AALFPA_042417_OR6 SICD**E**VMKPAIRFTTIVAVVNIVMG**M**AFTLLPIPEMIYHY**TIT**G**Q**WVRQLPFLIWWSFD**V** 180

.**.:****** :*::.****::**::**:*** ****:*: * ********* * **

(0)

CQUJHB000029_OR6 **L**SG**NK**YFL**V**YPLYIVIGFSGIIIHM**A**FDCLFCIL**T**AHIC**MN**FRIL**E**HD**F**EN**IKSAS**D**IN**E 239

AAEL017548_OR6 YSGFVYYFIYPLY**V**VIGFSGIIIHMGFDCLFCIL**S**AHLCVH**L**RILK**F**DLENLT**YG**LD**SS**E 240

AALFPA_042417_OR6 Y**A**GFVYYFIYPLYIVIGFSGIIIHMGFDCLFCIL**A**AH**I**CVHFRILKHDL**G**NLT**DL**LD**GT**E 240

:* *:::****:***********.********:**:*:::***:.*: *:. * .*

(2) (0)

CQUJHB000029_OR6 **TS**----E-**SIT**K**CLIH**VEKHQ**R**-L**HYKEAVDG**IF**S**F**T**L**C**YNFFVSS**I**IICIQGFM**I**TAAS 293

AAEL017548_OR6 N**M**---SIK**L**NPKLF**Y**IVEKHQ**N**ILECH**D**RMN**K**IFNFALFYNFFVSSFIICIQGFMVTAAS 297

AALFPA_042417_OR6 N**I**IENRKK**V**NPKLF**G**IVEKHQ**I**I**E**ECH**N**RMN**T**IFNFALFYNFFVSSFIICIQGFMVTAAS 300

. * : ***** . :: :: **.*:* *******:********:****

(0)

CQUJHB000029_OR6 GYTLIKFALFLASFLVELFLLCFYG**QDVS**ESS**SQ**VA**Q**AAYNCSWYN**ESKK**FR**QL**ILQ**I**I**M** 353

AAEL017548_OR6 GYTLIKFALFLASFLVELFLLCFYGHHIVESS**V**LVAEAAYNC**L**WYNTNHQFRTIILQM**V**N 357

AALFPA_042417_OR6 GYTLIKFALFLASFLVELFLLCFYGHHIVESS**I**L**A**AEAAY**H**CSWYNTNHQFRTI**V**LQMIN 360

*************************:.: *** .*:***:* *** .::** ::**::

(0)

CQUJHB000029_OR6 K**A**Q**R**PL**V**LMAWK**F**WPVN**IM**TF**S**SILSASWSYFTLLRTVY**YK** 394

AAEL017548_OR6 KGQ**I**PL**S**LMAWKIWPVNM**N**TFA**N**ILSASWSYFTL**I**RTVYAD 398

AALFPA_042417_OR6 KGQ**N**PL**T**LMAWKIWPV**D**M**S**TFASILSASWSYFTLLRTVYAD 401

*.* ** *****:***:: **:.***********:**** .

**OR8**

AAEL012254_OR8 MNDLVKFESFIRVPEIFFDMIGITRYGE**A**RDTWKARLKQAFFWSSYANTIFCLIIEHIYF 60

AALFPA_067679_OR8 MNDLVKFESFIRVPEIFFDMIGITRYGEPRDTWKARLKQAFFWSSYANTIFCLIIEHIYF 60

CQUJHB020308_OR8 MNDLV**R**FESFIRVPEIF**YG**MIGITRYGEPR**N**T**T**KARLKQ**L**FFWSSYANTIFCLIIEHIYF 60

*****:***********:.********* *:* ****** ********************

AAEL012254_OR8 IKAAGNFTNFLELTALAPCIGFTALSIVKIMTIKLNEAKLNGILDRLSDLFPRSHLDQDR 120

AALFPA_067679_OR8 IKAAGNFTNFLELTALAPC**M**GFTALSIVKIMTIKLNEAKLNGILDRLSDLFPRSH**Q**DQDR 120

CQUJHB020308_OR8 **FR**AAGNFTNFLELTALAPCIGFTALSIVKIMTIKLNEAKLNGILDRL**KE**LFP**VT**HL**E**QTR 120

::*****************:***************************.:*** :* :* *

AAEL012254_OR8 YRTYNYNLESQMVMKSFSILYMILIWIFNLLPLVSMLVNY**I**STG**I**LEKELPYFMWYWYDW 180

AALFPA_067679_OR8 YRTYNYNLESQMVMKSFSILYMILIWIFNLLPLVSMLVNYLSTGVLEKELPYFMWYWYDW 180

CQUJHB020308_OR8 YRT**HQ**YNLESQMVMKSFSILYMILIWIFNLLPLVSMLVNYL**MS**GVL**VR**ELPYFMWYWYDW 180

***::***********************************: :*:* :************

AAEL012254_OR8 HK**A**GYYEITFFHQNWGAFDSAVFNLSTDLLFCAIILLICLQFDILAYRLRHAKGDYKELE 240

AALFPA_067679_OR8 HK**V**GYYEITFFHQNWGAFDSAVFNLSTDLLFCAIILLICLQFDILAYRLRHAKGDYKELE 240

CQUJHB020308_OR8 H**RE**G**L**YEITFFHQNWGAFDSAVFNL**C**TDL**M**FCA**V**ILL**M**CLQFDI**I**AVRLRAAK**D**DP**Q**EL**I** 240

*: * ********************.***:***:***:******:* *** **.* :**

AAEL012254_OR8 **Q**CVKLHQSVVELSNQLEGIFSPSILVNFVGSSVIICLVGFQATS**N**ISAFDLFKFILFLIS 300

AALFPA_067679_OR8 **E**C**A**KLHQSV**I**ELSNQLEGIFSPSILVNFVGSSVIICLVGFQATS**D**ISAFDLFKFILFLIS 300

CQUJHB020308_OR8 **S**CV**Q**LHQ**T**VLEL**GD**QLE**S**IFSPSILVNF**L**GSSVIICLVGFQATS**Q**ISAFDLFKF**V**LFLIS 300

.*.:***:*:**.:***.**********:***************:*********:*****

(0)

AAEL012254_OR8 SLVQVFLLCYYGNKLIEASSQI**G**YCAFEGTWYMAD**L**RYQKSLLFVMTRAG**Q**WQKLTAMKF 360

AALFPA_067679_OR8 SLVQVFLLCYYGNKLIEASSQI**T**YCAFEGTWYMADVRYQKSLLFVMTRAGKWQKLTAMKF 360

CQUJHB020308_OR8 SLVQVFLLCYYGNKLIEASSQI**P**Y**A**AF**Q**G**E**WY**L**ADVRY**R**KSLLF**L**M**A**RAGKWQKLTAMKF 360

********************** *.**:* **:**:**:*****:*:***:*********

(0)

AAEL012254_OR8 SVVSLASYSAILSTSFSYFTLLKTIYEPSQK- 391

AALFPA_067679_OR8 SVVSLASYSAILSTSFSYFTLLKTIYEPK--- 389

CQUJHB020308_OR8 SVVSLAS**FTG**ILST**A**FSYFTLLKTIY**D**PSDSK 392

*******::.****:***********:*.

**OR10**

AAEL006003_OR10 --MASILDCPIVSVNARVWRFWSFVLKHDAMRYISIIPVTVMTFFMFTDLCRSWGNIQE**L** 58

AALFPA_080165_OR10 --M**E**SIL**N**CPIVSVNARVWRFWSFVLKHDAMRYISIIPVTVMTFFMFLDLG**H**SWG**DF**Q**D**V 58

CQUJHB000274_OR10 MT**A**A**P**ILDCPI**I**SVNVRVW**H**FWSFVLKHDAMRYISIIPV**G**VM**NV**FMF**A**DL**Y**R**A**WGNI**D**EV 60

**:***:***.***:******************* **..*** ** ::**:::::

(0) (0)

AAEL006003_OR10 IIKAYFAVLYFNAVLRTLILVKDRKLYENFMQGIS**N**VYFEIS**H**IDDH**K**IQSLL**K**SYTVRA 118

AALFPA_080165_OR10 IIK**G**YFAVLYFNAVLRTLILVKDRKLYENFM**E**GIS**KF**YFEIS**R**IDDH**Q**IQSLLRSYTARA 118

CQUJHB000274_OR10 II**N**AYFA**MIF**FNAVLRT**IFILCN**R**QD**YE**D**F**L**Q**R**I**AE**VY**S**EI**AM**IDDH**VV**Q**K**L**V**R**KF**T**K**RA 120

**:.***:::*******:::: :*: **:*:: *::.* **: **** :*.*::.:* **

AAEL006003_OR10 RMLSISNLALGAIISTCFVVYP**I**FTG**E**RGLPYGMFIPG**L**D**S**F**R**SP**H**YEIIYIVQVVLTFP 178

AALFPA_080165_OR10 RMLSISNLALGAIISTCF**T**VYP**M**FTG**V**RGLPYGM**Y**IPGVD**GYQ**SP**Q**YEIIYLVQVVLTFP 178

CQUJHB000274_OR10 R**L**LS**KA**NL**V**LGA**V**ISTC**Y**VVYP**L**FTG**T**R**S**LPYGMFIPGV**NN**F**KT**P**L**Y**Q**VF**FIG**Q**A**VLTFP 180

*:** :**.***:****:.***:*** *.*****:***::.:::* *::::: *.*****

AAEL006003_OR10 GCCMYIPFTSFFASTTLFGLVQIKTLQRQLQTFKD**N**I**S**S**QDKEK**V**K**TKV**V**KLIEDHKRII 238

AALFPA_080165_OR10 GCCMYIPFTSFF**V**STTLFGLVQIKTLQRQLQTFKD**G**I**G**S**HGNKNANLQ**V**I**KLI**Q**DHKRII 238

CQUJHB000274_OR10 GCCMYIPFTSFFA**T**TTLFGLVQI**Q**TLQRQL**R**TFKD**EVVKENRAL**V**ES**K**LE**K**C**IEDHKRII 240

************.:*********:******:**** : .... .: :: * *:******

(0)

AAEL006003_OR10 **T**YVSELNSLVTYICFVEFLSFG**M**MLCALLFLLNVIENHAQIVIVAAYIFMIISQIFAFYW 298

AALFPA_080165_OR10 **A**YVSELNSLVTYICFVEFLSFGLMLCALLFLLNVIENHAQ**M**VIVAAYIFMIISQIFAFYW 298

CQUJHB000274_OR10 **R**YVS**DV**NSLVTYIC**LI**EF**M**SFGLMLCALLFLLN**I**IEN**P**AQI**I**IV**V**AYIFMIISQIF**T**FYW 300

***::********::**:***:**********:*** **::**.***********:***

(0)

AAEL006003_OR10 HANEVREESMN**L**AEAAYSGPWVELD**N**SIKKKLLLIILRAQQPLEITVGNVYPMTLEMFQS 358

AALFPA_080165_OR10 HANEVREESMNIAEAAYSGPWVELDDSIKKKLLLIILRAQQPLEITVGNVYPMTLEMFQS 358

CQUJHB000274_OR10 HANE**L**REESM**G**IAEAAY**DA**PWVELDDS**M**KKKLLLII**A**RAQQPLEITVGNVY**A**MTLEMFQS 360

****:*****.:*****..******:*:******** ************** ********

AAEL006003_OR10 LLNASYSYFTLLRRVYN 375

AALFPA_080165_OR10 LLNASYSYFTLLRRVYN 375

CQUJHB000274_OR10 LLNASYSYFTLLRRVYN 377

*****************

**OR11**

AAEL011583_OR11 MQLKDEWIQEDDVYDNPLLRLTINGLKYYGILLYKSQPFKKLNCFRGVCFTASMLAFNVT 60

AALFPA_064092_OR11a MQLKDEWIQEDDVYDNPLLRLTINGLKYYGILLYKSQPFKKLNCFRGVCFTASMLAFNVT 60

AALFPA_060890_OR11b MQLKDEWIQEDDVYDNPLLRLTINGLKYYGILLYKSQPFKKLNCFRGVCFTASMLAFNVT 60

CQUJHB004338_OR11 M**E**LKDEWIQEDDVY**S**NPLLRLT**L**NGLKYYGILLYKSQP**L**KKLNCFRGVCFTASMLAFNVT 60

*:************.*******:***************:*********************

(1)

AAEL011583_OR11 QYVDLYQVWG**N**I**A**EMTANAATTLLFTTTIVRILHFYWNRARFNNAIKVADEGVQHLLRFG 120

AALFPA_064092_OR11a QYVNLYQVWGDISQMTANAATTLLFTTTIVRILHFYWNRARFNNAIKVADEGVQHLLRFG 120

AALFPA_060890_OR11b QYVNLYQVWGDISQMTANAATTLLFTTTIVRILHFYWNRARFNNAIKVADEGVQHLLRFG 120

CQUJHB004338_OR11 QYVDL**V**QVWGDI**G**EMTANAATTLLFTT**I**I**L**RI**F**HFYWNRARFNNAIK**F**ADEGV**R**H**I**L**DY**G 120

***:* ****:*.:************* *:**:**************.*****:*:* :*

AAEL011583_OR11 NAPEK**E**IFWDNV**K**YMNRLTAAFWICALVTANTMCVYALVQY**Q**SLKSMDSFNSTEPFDPPT 180

AALFPA_064092_OR11a NAPEKAIFWDNVRYMNRLTAAFWICALVTANTMCVYSLVQYHSLKSMEPFNSTKPFDPPT 180

AALFPA_060890_OR11b NAPEKAIFWDNVRYMNRLTAAFWICALVTANTMCVYSLVQYHSLKSMEPFNSTKPFDPPT 180

CQUJHB004338_OR11 **TPA**EKAIFWDNV**G**YM**K**RLTA**V**FWICALVTANTMCVYAL**IE**Y**C**S**VP**------**EA**E**RVE**PP**M** 174

. ** ****** **:****.***************:*::* *: .:: .:**

AAEL011583_OR11 ILRSWYPTDNI**VD**SF**A**TIYLIQLYIMYVGQLIVPCWHVFMVSLMLYARTALM**A**LNYKLAN 240

AALFPA_064092_OR11a ILRSWYPTDNIMESFTTIYLIQLYIMYVGQLIVPCWHVFMVSLMLYARTSLM**V**LNYKLAN 240

AALFPA_060890_OR11b ILRSWYPTDNIMESFTTIYLIQLYIMYVGQLIVPCWHVFMVSLMLYARTSLMILNYKLAN 240

CQUJHB004338_OR11 ILRSWYP**GGHKE**E**N**F**GA**IY**AV**QLYIMYVGQLIVPCWHVF**I**VSLM**V**Y**V**R**A**AL**T**ILN**H**KL**RH** 234

******* .: :.* :** :******************:****:*.*::* **:** :

AAEL011583_OR11 LE**Q**YAVSGMRSGRKIK**C**V**V**DPEEERCN**V**RKELIVECIQQQHKIFEYTRELEALTRGAMFM 300

AALFPA_064092_OR11a LEMYAVSG**L**RSGRKLKLVKDPEEERCNERKELIVECIQQQHKIFEYTRELEALTRGAMFM 300

AALFPA_060890_OR11b LEMYAVSGMRSGRKLKLVKDPEEERCNERKELIVECIQQQHKIFEYTRELEALTRGAMFM 300

CQUJHB004338_OR11 L**DA**Y**VT**SGMRSGR**L**I**REL**KDPEEER**L**N**A**R**R**ELI**I**EC**V**Q**R**Q**R**K**LH**EYT**D**ELE**S**L**IQ**G**PV**F**L** 294

*: *..**:**** :: : ****** * *:***:**:*:*:*:.*** ***:* :* :*:

(0) (0)

AAEL011583_OR11 DFVVFSVLLCALLFEASST**N**SFVQIFIDICYIMTMTA**M**LFLYYWHANEIHYQANLLSSSA 360

AALFPA_064092_OR11a DFVVFSVLLCALLFEASSTDSFVQIFIDICYIMTMTAILFLYYWHANEIHYHANLLSSSA 360

AALFPA_060890_OR11b DFVVFSVLLCALLFEASSTDSFVQIFIDICYIMTMTAILFLYYWHANEIHYHANLLSSSA 360

CQUJHB004338_OR11 DF**I**VFSVLLCALLFEAS**V**TDS**A**VQ**V**FID**V**CYIMTMTAILFLYYWHANEI**QH**Q**SDQ**LS**K**SA 354

**:************** *:* **:***:********:***********::::: **.**

(0)

AAEL011583_OR11 FMNDWYNYPRSVNRHLITFICYSNKPL**D**MKA**Y**IVSMSLDTFLAILRASYSYFTILKQAAG 420

AALFPA_064092_OR11a FMNDWYNYPRSVNRHLITFICYSNKPLNMKAFIVSMSLDTFLAILRASYSYFTILKQAAG 420

AALFPA_060890_OR11b FMNDWYNYPRSVNRHLITSICYSNKPLNMKAFIVSMSLDTFLAILRASYSYFTILKQAAG 420

CQUJHB004338_OR11 F**A**NDWYNYP**PK**VNR**N**L**LV**LICYS**I**KP**RI**MKAFIVSMSLDTF**I**AILRASYSYFTILKQAA**D** 414

* ******* .***:*:. **** ** ***:*********:*****************.

**OR13**

AAEL008368_OR13 MWQPLRKFLAPGPELLSFGLQMLRF**I**GLWGDRR**QV**VRYLLVL**F**SE**FI**FLIGPKALLGS**D**K 60

AALFPA_046408__OR13a MWPRLRKFLAPEPELLSFGLQMLRFVGLWGDRRRIVRYLLVLLSELMFLIGPKALLGSGK 60

AALFPA_064397_OR13b MWPRLRKFLAPEPELLSFGLQMLRFVGLWGDRRRIVRYLLVLLSELMFLIGPKALLGSGK 60

CQUJHB000018_OR37 ----**MSPAIP**P**DLQV**L**K**F**P**L**R**MLRFVGLWGDRR**EL**VRY**AS**V**V**L**CMSVV**LI**I**PKA**A**LGSGK 56

: : * ::*.* *:****:*******.:*** *::. :.** *** ***.*

AAEL008368_OR13 EGFDSTARNI**G**ELIFLVEVCISIGIFASRR**A**SFERL**IV**VLENILRRKWP**RN**LQDEI**Y**RFH 120

AALFPA_046408__OR13a EGIDSTVRNIAELIFLVEVCISIGIFASRRTSFERLMA**I**LEEILRRKWPQDLQDEIDRFH 120

AALFPA_064397_OR13b EGIDSTVRNIAELIFLVEVCISIGIFASRRTSFERLMAVLEEILRRKWPQDLQDEIDRFH 120

CQUJHB000018_OR37 **D**GFDS**F**ARNTAELIF**FT**EVC**V**SIGIFASRR**G**SFERL**VE**VL**R**E**TVLMYEDVE**L**LG**EI**AA**F**N** 116

:*:** .** .****:.***:********* *****: :*.: : :* .** *:

(2)

AAEL008368_OR13 RRMEFFARAYALYIGFLL**F**LY**N**CVP**I**GSTIVKL**I**RFDESERSDFMLVVELQFFWFDIRRN 180

AALFPA_046408__OR13a RRMEFFARAYALYIGFLVVLFCCVPVGSTLVKLVRFDESERSDFMLVVELQFFWFDIRRN 180

AALFPA_064397_OR13b RRMEFFARAYALYIGFLVVLFCCVPVGSTLVKLVRFDESERSDFMLVVEL-FFWFDIRRN 179

CQUJHB000018_OR37 R**K**MERF**SKS**YA**AW**IGF**W**VVLY**LGI**P**MIF**T**C**VK**V**V**FPG**E**GD**R**G**DFML**IA**ELQF**Y**W**L**DIRRN 176

*:** *:::** :*** :.*: :*: * **:: .*.:*.****:.** *:*:*****

AAEL008368_OR13 **VV**HYAIYM**A**FC**F**VAVSCSAYQS**T**LKGSVIVVVTQYGSKLFELISKRIDAM**RS**I**ERAA**DRD 240

AALFPA_046408__OR13a AFHYAIYMMFCLVAVSCSAYQSVLKGSIIVVVTQYGSKLFELISKRIEAMAKIPKQTDRD 240

AALFPA_064397_OR13b AFHYAIYMMFCLVAVSCSAYQSVLKGSIIVVVTQYGSKLFELISKRIEAMAKIPKQTDRD 239

CQUJHB000018_OR37 **LLD**YAIY**LV**FC**SM**A**IF**CS**S**YQS**T**LKG**A**VI**L**V**SI**QYG**T**KLFEL**VAMS**ID**RLGNVKEEIA**R**K** 236

..****: ** :*: **:***.***::*:* ***:*****:: *: : .: . *.

(0)

AAEL008368_OR13 RELREIVKLHG**M**A**L**EYV**Q**HLESTISFVMINQIMNCIFIWCLMMFYVSTNFGPNAANVMLL 300

AALFPA_046408__OR13a RELREIVKLHGLAMEYVHHLESTISFVMINQIMNCILIWCLMMFYVSTNFGPNAANVMLL 300

AALFPA_064397_OR13b RELREIVKLH**S**LAMEYVHHLESTISFVMINQIMNCILIWCLMMFYVSTNFGPNAANVMLL 299

CQUJHB000018_OR37 **NQ**LREIV**N**LH**K**LA**FQ**Y**TK**HLE**D**T**VC**F**M**MINQI**L**NCI**L**IWCLMMFYVSTNFGPNA**VC**V**II**L 296

.:*****:** :*::*.:***.*:.*:*****:***:*****************. *::*

(0)

AAEL008368_OR13 FLVLMGEMVVYCLNGTALSEQAAGVGHAIYNYPWY**K**ESVAMQK**N**MQL**M**IQRAQRPTGITA 360

AALFPA_046408__OR13a FLVLMGEMVVYCLNGTALSEQAAGVSHAIYHYPWYTESVQMQKHMQLIIQRAQRPTGVTA 360

AALFPA_064397_OR13b FLVLMGEMVVYCLNGTALSEQAAGVSHAIYHYPWYTESVQMQKHMQLIIQRAQRPTGVTA 359

CQUJHB000018_OR37 F**A**VLMGEM**IA**YC**V**NG**SK**L**A**E**T**AA**A**V**G**HA**V**Y**R**YPWY**N**E**PT**AMQK**D**MQLII**E**RAQ**K**PTGITA 356

* ******:.**:**: *:* **.*.**:*.****.* . ***.***:*:***:***:**

AAEL008368_OR13 AKFYFVNIERLGLV**T**QASYSYYLILK**N**RF 389

AALFPA_046408__OR13a AKFYYVNIERLGMVIQASYSYYLILKKRF 389

AALFPA_064397_OR13b AKFYYVNIERLGMVIQASYSYYLILKKRF 388

CQUJHB000018_OR37 AKFYFVNIERLGLV**V**QASYSYYLILKKRF 385

****:*******:* ***********:**

**OR15**

AAEL008448_OR15 MKYFEL**T**EPEA**A**MPLALRLLETYGLRGGKRKFLQFQVTILWE**L**L**M**IV**I**PKI**V**FGYRS-QD 59

AALFPA_076765_OR15 MKYFEL**V**EPEAVMPLALRL**M**ETYGLRGGKRKFLQFQVTILWE**F**L**T**IV**L**PKI**I**FGYRS-QD 59

CQUJHB006521_OR1 MK**FAP**L**QNRM**AVMP**FT**L**QY**L**RLF**GLRG**DR**R**NRVH**F**VLAL**L**YRV**L**LLNF**PK**LA**FG**F**R**D**R**I**D 60

**: * : *.**::*: :. :****.:*: ::* :::*:..* : :**: **:*. *

(1)

AAEL008448_OR15 LVIRGLSELLFQLHIMIRISIFAWHRFK**Y**E**S**L**ID**IIR**K**VYRKTFS**T**G**G**D**PTS**K**S**IIL**K**FN 119

AALFPA_076765_OR15 LVIRGLSELLFQLHIMIRISIFAWHRFKFE**D**L**VA**IIRRVYRKTFS**P**GADS**KL**K**E**IIL**D**FN 119

CQUJHB006521_OR1 LVIRSISELLFQ**I**HI**DL**R**AVL**FA**TKLRE**FE**E**L**AGLL**RKVY**N**K**VKTLD**ADS**PERK**II**EAS**N 120

****.:******:** :* :** : ::*.* ::*:**.*. : ..* :.** *

(2)

AAEL008448_OR15 **Q**MINKQSKGYFLYIMGCVSLF**S**VAPVVQSV**I**IF**MAN**Q**SR**NGT**E**KAEYVTMMEQEFYGLDI 179

AALFPA_076765_OR15 **K**MINKQSKGYFLYIMGCVSLF**T**VAPVVQSV**V**IF**ITH**QG**N**NGTDKAEYVTMMEQEFYGL**N**I 179

CQUJHB006521_OR1 **LG**IN**RR**SK**S**Y**A**LY**VAIA**V**TV**F**FWV**PVVQ**TTA**I**WLLNR**G**S**N**S**TD**RP**E**F**VTMME**L**EFYGLDI 180

**::**.* **: .*::* .****:. *:: ::. *.*:: *:***** *****:*

AAEL008448_OR15 RGNF**G**HY**AI**YV**A**LAGLAHYYSASFFAVTGV**I**IIC**G**VRCTILTFKLI**N**VRLSKLHELPK**Q**D 239

AALFPA_076765_OR15 RGNF**A**HY**VM**YV**G**LAGLAHYYSASFFAVTGVV**M**IC**A**VRCTILTFRLI**I**VRLSKLHELPK**E**D 239

CQUJHB006521_OR1 RGN**IW**HY**LV**Y**AS**L**SSV**AHYYSA**VY**FA**LS**G**M**VI**FSCIKSIAAL**F**E**L**VST**RL**AT**LHEL**SGKE** 240

***: ** :*..*:.:****** :**::*::::. ::. *.*: .**:.**** ::

(2) (0)

AAEL008448_OR15 IR**D**ELREIIDLHVDALRCIQLLEQIANLAMVIQI**I**DCVLIWISMILYMRNNLGVDAISLM 299

AALFPA_076765_OR15 IR**G**ELREIIDLHVDALRCIQL**M**EQIANLAMVIQIVDCVLIWISMILYMRNNLGVDAISLM 299

CQUJHB006521_OR1 **L**R**E**EL**ADLVE**LHV**NG**LRCI**E**LLE**N**I**N**NLAM**MV**Q**M**V**N**CVLIWISM**F**L**SIST**N**FTPEVV**SL**L** 300

:* ** ::::***:.****:*:*:* ****::*:::********:* : .*: :.:**:

(0)

AAEL008448_OR15 VLFVALTGETYALCDLLTQLTSESLAVTRAIIDCQWY**S**LPLDVQK**S**LSF**V**LFRAQR**K**EGI 359

AALFPA_076765_OR15 VLFVALTGETYALCDLLTQLTSESLAVTRAIIDCQWY**N**LPLDVQK**A**LSF**I**LFRAQR**N**EGI 359

CQUJHB006521_OR1 VL**LIVM**TGETY**V**LC**Q**L**A**T**E**L**SHVN**L**T**V**AES**I**HRSE**W**IQM**P**V**DVQK**G**L**AMM**L**Q**RAQ**KR**EG**L** 359

**::.:*****.**:* *:*: ..*:*:.:* .:* .:*:****.*:::* ***:.**:

AAEL008448_OR15 TAAKFF**F**MDIERFG**S**VAQTSYSIYVVLKDQL 390

AALFPA_076765_OR15 TAAKFFYMDIERFG**N**VAQTSYSIYVVLKDQL 390

CQUJHB006521_OR1 TAAKFFYMD**V**ERFG**R**VAQTSYSI**FI**VLK**ERI** 390

******:**:**** ********::***:::

**OR31**

AAEL013217_OR31 --------------------------**M**A**PTQNGRDRE**KFLRVQLLCLALIGI**K**RHETVSS 34

AALFPA_070001_OR31a -------------------MALPSPTPADGRRTT**S**QGKFLRVQLLCLALIGVQRLETVSS 41

AALFPA_067456_OR31b -------------------MALPSPTPADGRRTT**S**QGKFLRVQLLCLALIGVQRLETVSS 41

AALFPA_062046_OR31c -------------------MALPS**S**TP**V**DGRTTT**A**QGKFLRVQLLCLALIGVQR**D**ETVSS 41

CQUJHB008764_OR95 MA---------TFTSKIAW**T**A**NDGNHRRGRSPDKEKD**NFLRVQLF**T**MAL**N**GI**RK**HETVSS 51

CQUJHB011798_OR96 MATTTAKATSNPGTPRAAW**VS**-----**DVG**GRT**IDDEE**NFLRVQLF**S**MALIGVQ**ER**ETV**P**S 55

. :******: :** *::. *** *

AAEL013217_OR31 R**T**IFHVCFISMVIMDLATILFALEHANDIALVCDCLGPTFTAYLGIVKQYCLSAHRVELW 94

AALFPA_070001_OR31a RAIFHVCFISMVIMDLATILFALEHANDIALVCDCLGPTFTAYLGIVKQYCLSAHRVELW 101

AALFPA_067456_OR31b RAIFHVCFISMVIMDLATILFALEHANDIALVCDCLGPTFTAYLGIVKQYCLSAHRVELW 101

AALFPA_062046_OR31c RAIFHVCFISMVIMDLATILFALEHANDIALVCDCLGPTFTAYLGIVKQYCLSAHRVELW 101

CQUJHB008764_OR95 RIYFY**G**CF**LT**MLIMDLA**G**V**H**FA**Y**Q**N**AG**E**I**L**LVCDCLGPTFT**CF**LG**V**VKQYYL**DV**HR**E**QLW 111

CQUJHB011798_OR96 RIY**S**Y**F**CF**Y**S**L**LIMDL**SM**VLFA**V**QH**F**GD**MV**LVCDCLGP**G**FTAYLG**M**VKQ**H**YLS**EQ**R**K**QLW 115

* : ** :::****: : ** :: .:: ******** **.:**:***: *. :* :**

(1)

AAEL013217_OR31 NIIETLRRLKDYAGTSEIESIERNNKIDRFLATAYL**M**SASATGSLFI**I**AALAKG**C**YKLIF 154

AALFPA_070001_OR31a NIIETLRRLKDYAQADEIVSIERNNKIDRILATAYLVSASATGSLFIFAALAKGFYKLIF 161

AALFPA_067456_OR31b NIIETLRRLKDYA--DEIVSIERNNKIDRILATAYLVSASATGSLFIFAALAKGFYKLIF 159

AALFPA_062046_OR31c NIIETLRRLKDYAQADEIVSIERNNKIDRILATAYLVSASATGSLFIFAALAKGFYKLIF 161

CQUJHB008764_OR95 **Y**II**HE**LRKLKQ**N**ATVS**D**IE**M**IEKNNKID**Q**FLATAY**FA**S**SMG**TG**TI**FIV**E**AILKGTY**NYFI** 171

CQUJHB011798_OR96 **E**II**YA**L**K**KLKQ**I**AKPDEI**R**SIERNN**Q**IDR**Y**LATAYL**T**SA**VI**TG**SH**FIV**T**AIVK**AV**Y**SKVV** 175

** *::**: * .:* **:**:**: *****: *: **: **. *: *. *. ..

(2)

AAEL013217_OR31 QN**I**IEWG**F**PLSLSFPF**K**TSHPI**V**F**G**VFFVWSSAAIYIVVFCSVSSDASFGGLASNVVVHF 214

AALFPA_070001_OR31a QNSIEWGLPLSLSFPFNTSQPIIFALFFIWSSAAIYIVVFCSVSSDASFGGLASNVVVHF 221

AALFPA_067456_OR31b QNSIEWGLPLSLSFPFNTSQPIIFALFFIWSSAAIYIVVFCSVSSDASFGGLASNVVVHF 219

AALFPA_062046_OR31c QNSIEWGLPLSLSFPFNTSQPIIFALFFIWSSAAIYIVVFCSVSSDASFGGLASNVVVHF 221

CQUJHB008764_OR95 **R**N**C**IEW**N**LP**IAI**SFPFDISHP**A**IFA**F**FFIW**C**SAATYMVVF**S**SVSSDAGFGGLASNLVVHF 231

CQUJHB011798_OR96 **HGKFV**W**Q**LPL**LQ**S**Y**PFDISHP**LM**FAV**L**F**V**W**T**SA**T**IYMVVF**G**SVSSDA**A**FGGLASNLVVHF 235

:. : * :*: *:**. *:* :*..:*:* **: *:*** ******.*******:****

AAEL013217_OR31 KLLQKRLQD**A**TF**A**DNDENLK**Q**LIEYHSLLL**N**LSRK**I**MSSFRVIIINNLLVASVLLCVLGF 274

AALFPA_070001_OR31a KLLQKRLQDTTFTDNDENLKYLIEYHSLLLGLSRKLMSSFRLIIINNLLVASVLLCVLGF 281

AALFPA_067456_OR31b KLLQKRLQDTTFTDNDENLKYLIEYHSLLLGLSRKLMSSFRLIIINNLLVASVLLCVLGF 279

AALFPA_062046_OR31c KLLQKRLQDTTFTDNDENLKYLIEYHSLLLDLS**Q**KLMSSFRVIIINNLLVASVLLCVLGF 281

CQUJHB008764_OR95 K**I**LQ**N**RF**K**DR**R**FED**D**D**Q**SL**E**DLIEYH**T**LVL**K**LSRRLMSSFR**I**IILNNLLVASVLLCVLGF 291

CQUJHB011798_OR96 K**FI**Q**AG**F**R**DR**S**FEDND**P**SLKDLIEYH**RH**VLDLSRKL**I**S**AY**R**P**I**ML**NN**FI**VAS**F**LLCVLGF 295

*::* ::* * *:* .*: ***** :* **::::*::* *::**::***.*******

(0)

AAEL013217_OR31 QLVMFLGSTLMLIY**L**MYVTAIVIQITFFAYYGSLLSHESE**E**V**SSS**IYCSNWYEASPKTRR 334

AALFPA_070001_OR31a QLVMFLGSTLMLIYFMYVTAIVIQITFFAYYGSLLSHESEAVGNAIYCSNWYEATPKTRR 341

AALFPA_067456_OR31b QLVMFLGSTLMLIYFMYVTAIVIQITFFAYYGSLLSHESEAVGNAIYCSNWYEATPKTRR 339

AALFPA_062046_OR31c QLVMFLGSTLMLIYFMYVTAIVIQITFFAYYGSLLSHESEAVGNAIYCSNWYEATPKTRR 341

CQUJHB008764_OR95 **EM**V**VY**LG**TS**LMLLYI**T**Y**I**TAIVIQI**F**FFSYYGSQL**LY**ESAAVGDAIYCSNWYEATPKTR**K** 351

CQUJHB011798_OR96 QLV**L**F**M**GST**M**M**F**LYI**VF**VTAIVIQITFFSYYGSQLSHESA**L**VGDAIYCSNWYE**T**SPKTRR 355

::*:::*:::*::*: ::******* **:**** * :** *..:********::****:

(0)

AAEL013217_OR31 ILLQCLMRAQVPVNTKAGFMVASLPTLRAILNSAGSYVALLLSFTDN 381

AALFPA_070001_OR31a ILLQCLMRAQVPVNTKAGFMVASLPTLRAILNSAGSYVALLLSFTD- 387

AALFPA_067456_OR31b ILLQCLMRAQVPVNTKAGFMVASLPTLRAILNSAGSYVALLLSFTD- 385

AALFPA_062046_OR31c ILLQCLMRAQVPVNTKAGFMVASLPTLRAILNSAGSYVALLLSFTD- 387

CQUJHB008764_OR95 LLL**F**C**IK**RAQVPVNTKVG**I**MVASLPTFRAI**V**NSAGSYVALLLSFT**E**D 398

CQUJHB011798_OR96 LLLQCLMRAQVPVN**IR**VGF**IE**AS**M**PTFRAILNSAGSYVALLLSFTDT 402

:** *: ******* :.*:: **:**:***:**************:

**OR71**

AAEL017564_OR71 MELS**YH**RSLP**P**ELQIMPFQLRCMELIGL**I**GPKGRFYRFVLAFGWGTFVILLPKSVLGIGS 60

AALFPA_064397_OR71 MELS**HY**RSLP**S**ELQIMPFQLRCMELIGL**A**GPKGRFYRFVLAFGWGTFVILLPKSVLGIGS 60

****::**** ***************** *******************************

(1)

AAEL017564_OR71 **SE**LDAIIKGFAELLFEGNLFIAVASL**V**PKLPL**V**KRLLHVLSEIF**R**Q**A**T**H**D**K**RD**V**KD**H**CY**A** 120

AALFPA_064397_OR71 **PR**LDAIIKGFAELLFEGNLFIAVASL**I**PKLPL**L**KRLLHVLSEIF**T**Q**V**T**R**D**A**R-**S**KD**Q**CY**E** 119

.************************:*****:*********** *.*:* * **:**

AAEL017564_OR71 LICEQNSKIDKFCKFYFIYCCFGPFVFCIPAMVTSYVRYFG**TTN**NGT**NA**---N**GS**E**H**L**R**F 177

AALFPA_064397_OR71 LICEQNSKIDKFCKFYFIYCCFGPFVFCIPAMVTSYVRYFG**GAG**NGT**DN**ADSN**ET**E**Q**L**L**F 179

***************************************** :.***: * :*:* *

(2)

AAEL017564_OR71 ELPMEQEFY**W**L**P**IRTNFA**C**YHLFL**TL**SLSAYCVCSYMSVIKVSTLLIMIKY**C**SL**V**YRLVA 237

AALFPA_064397_OR71 ELPMEQEFY**G**L**Q**IRTNFA**H**YHLFL**AA**SLSAYCVCSYMSVIKVSTLLIMIKY**S**SL**A**YRLVA 239

********* * ****** *****: *************************.**.*****

(0) (0)

AAEL017564_OR71 IRIR**E**L**GK**LP**PGRKQDD**----TD**EDERT**KM**VK**VKEVV**EM**H**E**KALE**A**TDLVE**KV**INIPIAM 293

AALFPA_064397_OR71 IRIR**K**L**AE**LP**ASGQEQN**FEEVTD**GGDVL**KM**SM**VKEVV**DL**H**K**KALE**V**TDLVE**EI**INIPIAM 299

****:*.:** . :::: ** .: ** *****::*:****.*****::*******

(0)

AAEL017564_OR71 QF**M**ACILFWCMTM**V**YVSTNINFNLFNVMVLFWLSLIETYGYSYLGTEL**S**E**D**AK**A**VG**HAV**Y 353

AALFPA_064397_OR71 QF**I**ACILFWCMTM**F**YVSTNINFNLFNVMVLFWLSLIETYGYSYLGTEL**T**E**E**AK**T**VG**QVI**Y 359

**:**********.**********************************:*:**:**:.:*

AAEL017564_OR71 **D**LPWYEDSAQLQRYYRLMIQR**S**QQN**I**GVTAAKFFIVGIEKFGKVVNLSYSYYLVLKDVLD 413

AALFPA_064397_OR71 **E**LPWYEDSAQLQRYYRLMIQR**T**QQN**T**GVTAAKFFIVGIEKFGKVVNLSYSYYLVLKDVLD 419

:********************:*** **********************************

AAEL017564_OR71 **R**L 415

AALFPA_064397_OR71 **S**L 421

*
